# Lipid Biomimetic Models as Simple Yet Complex Tools to Predict Skin Permeation and Drug–Membrane Biophysical Interactions

**DOI:** 10.3390/pharmaceutics16060807

**Published:** 2024-06-14

**Authors:** Eduarda Fernandes, Carla M. Lopes, Marlene Lúcio

**Affiliations:** 1 CF-UM-UP—Centro de Física das Universidades do Minho e Porto, Departamento de Física, Universidade do Minho, 4710-057 Braga, Portugal; 2 FFP-I3ID—Instituto de Investigação, Inovação e Desenvolvimento, FP-BHS—Biomedical and Health Sciences Research Unit, Faculdade de Ciências da Saúde, Universidade Fernando Pessoa, 4200–150 Porto, Portugal; cmlopes@ufp.edu.pt; 3 UCIBIO—Applied Molecular Biosciences Unit, MedTech–Laboratory of Pharmaceutical Technology, Faculty of Pharmacy, University of Porto, 4050-313 Porto, Portugal; 4 Associate Laboratory i4HB, Institute for Health and Bioeconomy, Faculty of Pharmacy, University of Porto, 4050-313 Porto, Portugal; 5 CBMA—Centro de Biologia Molecular e Ambiental, Departamento de Biologia, Universidade do Minho, 4710-057 Braga, Portugal

**Keywords:** *Stratum Corneum*, SC lipid model systems, SC surrogate, in vitro skin permeation

## Abstract

The barrier function of the skin is primarily determined by its outermost layer, the *Stratum Corneum* (SC). The SC consists of corneocytes embedded in a lipid matrix composed mainly of ceramides, cholesterol, and free fatty acids in equimolar proportions and is organised in a complex lamellar structure with different periodicities and lateral packings. This matrix provides a diffusion pathway across the SC for bioactive compounds that are administered to the skin. In this regard, and as the skin administration route has grown in popularity, there has been an increase in the use of lipid mixtures that closely resemble the SC lipid matrix, either for a deeper biophysical understanding or for pharmaceutical and cosmetic purposes. This review focuses on a systematic analysis of the main outcomes of using lipid mixtures as SC lipid matrix models for pharmaceutical and cosmetic purposes. Thus, a methodical evaluation of the main outcomes based on the SC structure is performed, as well as the main recent developments in finding suitable new in vitro tools for permeation testing based on lipid models.

## 1. Introduction

The skin is the largest organ of the body, and its primary function is to protect the body against invasion by exogenous substances, including dust, pathogens, compounds, and ultraviolet radiation [1,2,3]. This protective function is pivotal in diverse fields of research, such as toxicology and risk assessment and pharmaceutical and cosmetic products development. The major barrier function of the skin is located in the *Stratum Corneum* (SC), the outermost layer of the human skin, which is composed of corneocytes embedded in a matrix of lipid multi-layered lamellar structures known as the intercellular lipid matrix (ILM). This ILM is rich in ceramides (Cers) (40–50%), cholesterol (Chol) (20–25%), free fatty acids (FFAs) (15–25%), and a lesser quantity of cholesterol sulfate (ChS) (5–10%) that are self-associated in multilayers [2,4,5]. Corneocytes are grouped in clusters separated by micron-sized channels [6]. The lipids in the intercorneocytes are assembled in parallel and as repeated bilayers, i.e., head-to-head and tail-to-tail [7]. The SC’s lipid structure and composition differ from that of other biological membranes that comprise crystalline phospholipids. SC has two lamellar phases with repeated distances of 5–6 and 12–14 nm [7]. The chemical composition and the structural organisation of SC may be defined as a “brick and mortar” model, where the “bricks” are the cells and the “mortar” is the ILM [8], both conferring resistance to skin permeation. Most topically applied compounds are unable to permeate the SC due to its corneocyte-rich nature, and consequently, the transport through the skin predominantly occurs via passive diffusion through the ILM. The passive diffusion of bioactive compounds across this lipid lamellar structure implies a tortuous movement between corneocytes through interlamellar regions of the ILM (i.e., tail–tail region of intercorneocyte lipids) [6,7,9]. Additionally, the SC presents high density and low hydration (15–20%) compared to the body’s average 70% hydration. Therefore, the SC acts as a barrier for the diffusion of the majority of compounds, specifically the ones that present high molecular weight (>500 Da) and inadequate solubility [2,10,11]. A non-linear pH gradient between the upper and lower regions of the SC, which may range between 4.5 on the surface and 7.0 at the lower regions, can also limit the diffusion through the skin. Additionally, other important physiological conditions, such as temperature, as well as the presence of enzymes, can influence the rate of skin permeation [12,13,14,15].

Despite the numerous benefits that the skin provides as a route of administration for several compounds, the permeation of molecules via skin layers remains a challenge because of the significant variability between people’s skin and between skin sites on an individual [4,7,16], as well as the skin barrier’s effectiveness in limiting the type and amount of compound that can permeate it (mainly its outermost layer of epidermis, the SC) [7,17]. Therefore, estimating skin permeation rates is crucial in pharmaceutical and cosmetic research to hasten the development of dermal or transdermal compounds and/or formulations. However, estimating the permeability of a specific compound through the skin is often difficult due to the very complex nature of the different structures and mechanisms that comprise the permeation pathway [17]. Skin permeation studies are carried out for two main reasons: to study the impermeability of the skin to toxic chemicals, allergens, and irritants or to evaluate the ability of a drug/compound, commonly carried in a delivery system, to permeate the skin barrier and be delivered to a particular skin layer or even directly into the bloodstream. In this context, different skin models have been developed, ranging from in vitro to in vivo [18], from the simplest to the most complex.

Conventional in vitro skin permeation studies typically employ the Franz diffusion cell, a device that sandwiches a skin surrogate between donor and receptor compartments. Ideally, human skin would be the gold standard for such investigations to ensure an accurate representation of the processes involved, but its complex nature and limited availability, as well as the inter- and intra-individual variability and ethical constraints, hinder its widespread use in the early stages of compounds and/or formulation research [19,20,21,22]. As a result, animal skin surrogates are often employed, albeit with significant anatomical disparities and non-standardised protocols leading to inconsistent permeability data [23,24,25,26]. Moreover, with the European Union (EU) Cosmetic Regulation (EC 1223/2009) [27] imposing constraints on products involving animal testing in the EU, as well as the EU REACH regulation (Registration, Evaluation, Authorization and Restriction of Chemicals) [28] recommending/requiring the use of alternatives to animal assays [29,30], there is a clear and urgent need for efficient, precise, and cost-effective models to be used in the early stages of drug/compound research. In this regard, artificial model membranes based on lipid mixtures are attracting increasing attention. Since the 1990s, these lipid mixtures that mimick the SC ILM have been employed for various purposes, including biophysical characterisation and, more recently, interaction and permeation profiling for cosmetic and pharmaceutical applications. While the literature includes reviews on the use of lipid models for biophysical SC ILM characterisation [31,32,33,34,35], none of them focus on the development of these models into a new generation of skin diffusion and permeation tools. This review aims to bridge this gap by providing an overview of the main outcomes of biophysical SC ILM characterisation through lipid models and delving into their transformation into innovative lipid-based SC surrogates (SCS). The described SCS hold the promise of revolutionising in vitro studies in pharmaceuticals and cosmetics, offering a powerful alternative to traditional skin models for diffusion and permeation investigations.

## 2. Composition and Molecular Assembly of the *Stratum Corneum* Lipid Matrix

The human ILM is mainly composed of mixtures of Cers, Chol, and FFAs. Cers are sphingolipids consisting of a fatty acyl chain (resultant from a fatty acid) amide-linked to a sphingoid base. According to the type of sphingoid base, different nomenclatures are attributed to Cers: sphingosine (S), dihydrosphingosine (dS), phytosphingosine (P), and 6-hydroxysphingosine (H). The fatty acyl chain has also different nomenclatures with two to four hydroxyl (OH) functional groups and a monosubstituted amide group (N–C=O), which behave as hydrogen bond donors and acceptors [36] and can be non-substituted (N), α-hydroxylated (A), ω-hydroxylated (O), or bearing the ω-linoleyloxy group [37]. The combination of these letters designates the type of fatty acyl and sphingoid base in the Cers nomenclature. The structural variation of the long acyl chain linked to the sphingoid base via an amide linkage leads to a broad diversity of Cers, with around 17 subclasses of Cers identified in human skin [38,39]. In the native SC lipid matrix, Cers have acyl chains that can range from long (C13 to C19), very long (C20 to C26), or ultralong (˃C28) [40], and FFA’s chain lengths can range from C16 to C30, with a predominance of C24 and C26 (33.7 and 25.2%, respectively) [41].

**Figure 1:**
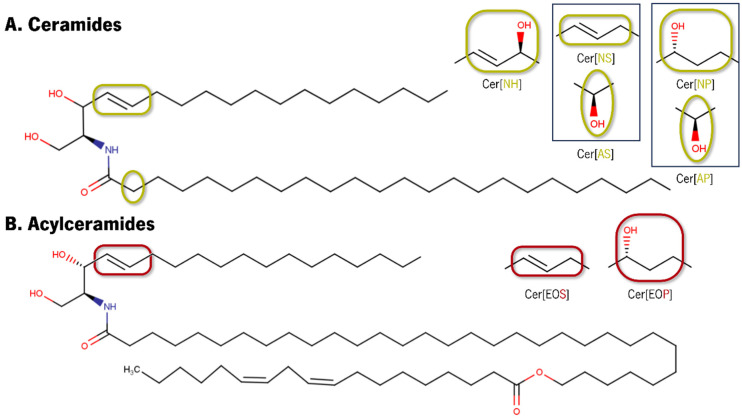
Schematic illustration of the main differences at the Cers headgroup level. Chemical structures were drawn in MarvinSketch^®^ version 5.3.1.

In Figure 1 are depicted the main structural differences between Cer headgroups.

Cer[AP] and Cer[NP] differ slightly at the headgroup level, with Cer[NP] featuring three OH groups and Cer[AP] having four OH groups [42].

Although the function of each Cer class has been the subject of intense debate and scrutiny, some consensus can be found in the literature. Considering the skin barrier function, while acylCers (Cer[EOS], Cer[EOP], and Cer[EOH]) are of pivotal importance due to their really long fatty acid chains [43,44], both classes of α-hydroxylated acyl chains (Cer[AS], Cer[AdS], Cer[AP], and Cer[AH]) and non-hydroxy fatty acid Cers (Cer[NdS], Cer[NH], Cer[NP], and Cer[NS]), with their involvement in intermolecular hydrogen bonding interactions, provide structural cohesion [45,46].

## 3. *Stratum Corneum* Lipid Model Membranes

**Figure 2:**
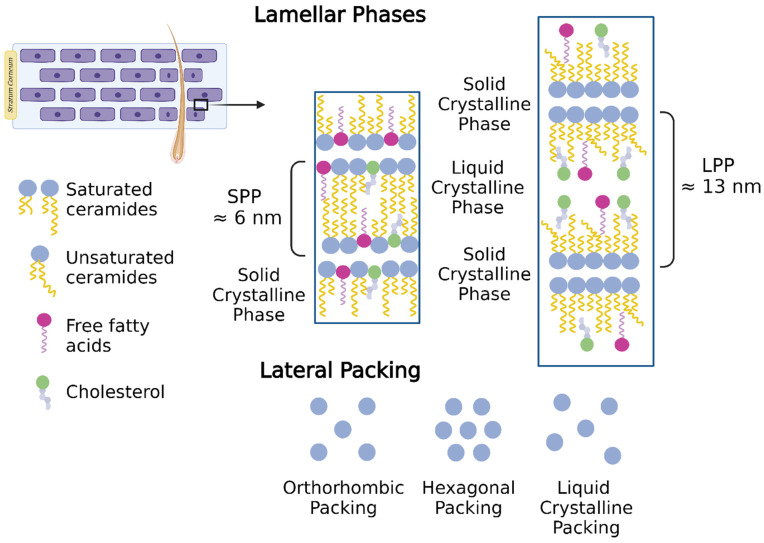
Lipids constituting the intercellular lipid matrix (ILM) of the *Stratum Corneum* (SC) are assembled in two crystalline coexistent lamellar phases: a short periodicity phase (SPP) and a long periodicity phase (LLP) and can be laterally packed in orthorhombic, hexagonal, or liquid-like unit cells (created with BioRender.com).

Due to structural differences in Cers—acyl chain length and mobility—SC lipids are assembled in two lamellar phases: a short periodicity phase (SPP) with a repeated distance of ≈5–6 nm and a long periodicity phase (LLP) with a repeated distanced of ≈12–14 nm (Figure 2) [47]. Regarding lateral packing, at the human skin surface temperature (≈32 °C), the SC lipids are mainly assembled in an orthorhombic packing, while a low level of lipids follow hexagonal or liquid crystalline packing (Figure 2) [47].

Despite their simplicity, SC lipid models can reproduce the characteristics of native SC lipid matrix assembly [48]. Numerous techniques, particularly X-ray diffraction, neutron diffraction, infrared (IR) spectroscopy, and nuclear magnetic resonance (NMR), have been employed to extensively decipher the structure of SC ILM through lipid mixtures of synthetic or natural lipids. Understanding the SC ILM structure is paramount to develop models that can mimick the SC barrier function in skin permeation studies. The most recent literature reporting examples of SC lipid model mixtures from 2010 onwards is presented in Table 1, with older studies [30,43,44,45,46,48,49,50,51,52,53,54,55,56,57,58,59,60,61,62,63,64,65,66,67,68,69,70,71,72,73,74,75,76,77,78,79,80,81,82,83,84,85,86,87,88,89,90,91,92,93,94,95,96,97,98,99,100,101,102,103] reviewed in the Supplementary Materials (Table S1).

As detailed in Table 1, both natural and/or synthetic Cers can be employed in SC mimetic mixtures. Natural Cers can be isolated from different human/animal sources, which is indicated by the subscript prior to Cer nomenclature: pig (_pig_Cer), human isolated (_h_Cer), or bovine brain (_BB_Cer). While acyl chain lengths vary greatly in natural Cers, synthetic Cers have well-defined chain lengths. Synthetic forms are more prone to be affected by the FFA composition, and its use in general leads to reduced repeated distances [49]. Multiple attempts have been dedicated to unravel the minutiae of the SC ILM structure, as evidenced by the extensive literature on this subject. Although SC lipid models for studying compounds interactions with the SC lipid matrix are also presented in Table 1, this review will mainly focus on the structural characteristics of such models.

The lamellar coexistence of two different periodicities, i.e., SPP and LPP, is consensual; however, their spatial assembly remains unclear, and several models have been proposed (see [34] for a detailed review of the structural organisation of SPP and LPP). Notwithstanding, the most widely accepted models in the literature can be assigned to the sandwich model for LPP and the armature reinforcement model as an extension of the sandwich model for the SPP. Therefore, both periodicity phases and their most widely accepted assembly model will be detailed in the next sections.

### 3.1. The Long Periodicity Phase (LPP)

**Figure 3:**
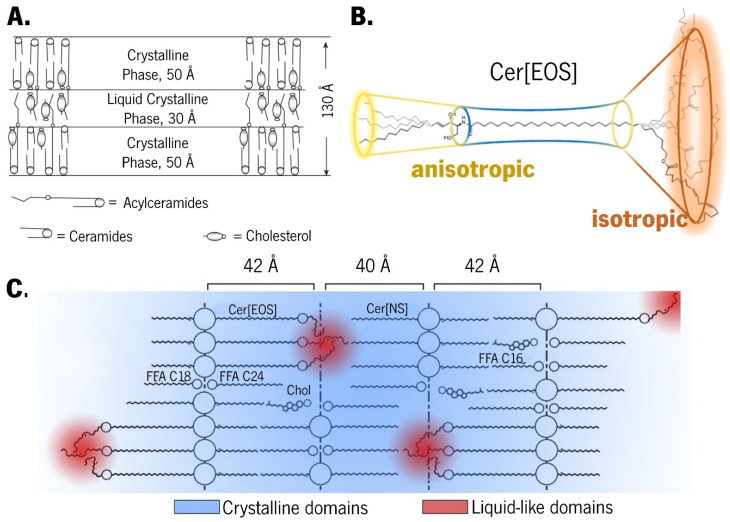
(**A**) Schematic illustration of the sandwich model purposed for a long periodicity phase (LPP) structure. Adapted with permission from [162]. Copyright 2007, Springer Nature. (**B**) Representation of the shape of a Cer[EOS] molecule. Adapted with permission from [161]. Copyright 2023, Elsevier. (**C**) Depiction of the suggested locations of the liquid-like domains in the LPP structure. Adapted with permission from [139]. Copyright 2018, Elsevier.

The sandwich model was originally proposed for LPP based on X-ray diffraction data obtained from isolated human skin. This model suggests a ternary lamellar structure with two solid crystalline layers surrounding a more fluid liquid crystalline core (Figure 3A) [43,50,162,163]. The unique backbone of Cer[EOS], which displays both rigid and mobile regions, has a pivotal role in the formation of this ternary lamellar structure due to its flexible segments at terminal regions, rigid middle segments near the carboxyl groups, and linoleate segments demonstrating rapid isotropic reorientation but slow self-diffusion (Figure 3B) [50,134,136,153,157,161]. These isotropic fluid chains are a fundamental and essential feature of the LPP structure in the context of the sandwich model [136].

Considering that the Cer[EOS] headgroup is anchored in the lamellar interface of the LPP structure and that linoleate chains are covalently bonded to the remaining rigid regions of the molecule preventing translational diffusion, a less dense packing at the end of the molecule or in the middle of the lipid layer would allow for isotropic reorientation [136]. The inner liquid crystalline phase layer has been observed even in the absence of water, and it is attributed to the steric confinement of the linoleate chains of Cer[EOS], which results in local hydrocarbon nanodroplets (Figure 3C) [51,139,161]. Indeed, the unsaturation level has an active role on the LPP formation, as observed by the lack of LPP as a consequence of replacing Cer[EOS]-linoleate by the -stearate form [157]. Similarly, the proportion of lipids forming LPP was reduced to a greater extent in _h_Cer:Chol mixtures in which _h_Cer[EOS] was replaced by a synthetic form of Cer[EOS]-linoleate as opposed to _h_Cer[EOS]-oleate mixtures [50]. De Sousa Neto et al. studied the role of different unsaturation degrees and found that, in the presence of Cer[EOS], even at high temperatures, the stearate moiety stabilised the interchain coupling, and the LPP formation was only possible in the presence of a specific level of unsaturation, such as Cer[EOS]-linoleate and -oleate moieties [157]. Moreover, the high degree of mobility at C18, along with conformational disordering and/or folding of the linoleate moiety in the inner headgroup regions, compensates the long chains of Cers and FFAs from opposite directions [159]. Therefore, as longer chain lengths lead to enhanced van der Waals interactions and reduced interchain distances [49], Cer[EOS] with its elongated acyl chains acts as a molecular rivet, sticking together the opposing lipid sheets and reinforcing the LPP structure [134,162]. Notwithstanding, the lack of a multilamellar structure in a Cer[EOS]:Chol:PA mixture [52] leads to investigations on other pivotal factors affecting the LPP formation. Several evidence suggesst that the presence of other Cers alongside Cer[EOS] contributes to LPP formation. In a mixture containing 15 mol% Cer[EOS] and 85 mol% Cer[NS], along with Chol and FFA, the coexistence of LPP and SPP predominantly displaying an orthorhombic packing was observed. However, increasing the Cer[EOS] concentration to 40 mol% resulted in exclusive LPP formation [146]. Consequently, the optimal molar ratio of Cer[EOS]:Cer[NP]:_BB_Cer[EOH] as the Cer component for the formation of LPP in a lipid mixture was investigated, and the authors found that, aside from a low sensitivity towards changes in the component ratio, the optimal fraction on the Cer component was indeed dependent on the presence or absence of FFAs [53]. Although Cer[EOS] seems to contribute to stabilise the lateral orthorhombic packing in the centre of the lamellae by increasing the lipids forming this packing lattice on the LPP [157], there is also a pivotal role of FFA in governing the lateral packing of the lipid mixtures, since both orthorhombic and hexagonal lateral packing are required for LPP formation [157,164]. The addition of long-chain FFA into a mixture of _h_Cer:Chol induced a transition from a hexagonal to an orthorhombic packing [50], whereas the FFA presence, even at low concentrations, lead to the formation of orthorhombic lateral packing, and in its absence, only hexagonal packing was observed [54]. However, although LPP formation occurred, low FFA levels led to a considerable amount of Chol-phase separating, while a higher FFA content led to decreased phase separation without affecting the LPP formation [159]. This was also observed in a mixture of _h_Cer:Chol:FFA, in which varying the FFA proportions revealed a distribution of Cer and FFA into two lamellar phases, with a minor portion of Chol separated into solid crystalline domains [54]. Furthermore, other mixtures employing _h_Cer with Chol, FFA, and ChS in varying ratios were also investigated [51]. In the absence of FFA and ChS, the mixture predominantly displayed a LPP phase with hexagonal lateral packing, while a small fraction of the lipids formed a fluid phase. However, in the absence of _h_Cer[EOS], only a minor portion of the lipids formed LPP, with SPP predominating [51]. When FFA was added to the mixture containing the whole _h_Cer, it revealed a SPP supremacy, which was predominantly orthorhombically packed [51]. This is in line with another study that reported that, although an initial increase in the relative amount of FFA or Cer[EOH] promoted LPP formation, when an optimal amount of Cer:FFA components was exceeded, it led to reduced LPP and a shift toward SPP dominance [49].

Investigations using _pig_Cer have revealed both similarities and differences between _h_Cer- and _pig_Cer-containing systems. Common aspects include LPP dominance and hexagonal packing in _h_Cer:Chol mixtures, consistent phase behaviour despite Cer:Chol ratio adjustments, a transition from hexagonal to orthorhombic packing upon FFA addition, increased Chol miscibility due to a ChS presence, and a significant reduction in the LPP phase when Cer[EOS] was absent. On the other hand, the primary differences were observed in the liquid lateral packing of _h_Cer:Chol:FFA only upon ChS addition; the addition of FFA not only induced an orthorhombic transition but also decreased the proportion of lipids forming LPP in _h_Cer mixtures, and larger fractions of Chol could be intercalated into lamellar phases of _h_Cer compared to _pig_Cer mixtures [51]. These differences were assigned to the documented association between the proportion of lipids forming fluid phases [50], with the fraction of Cer containing linoleic acid linked to a ω-hydroxy fatty acid being higher in the _h_Cer than in _pig_Cer [55].

These studies lead to other critical factors affecting the LPP formation, in which all the mixture components have specific roles that must be balanced. The presence of Chol has been also demonstrated to be required for both LPP formation and to increase the packing density within the LPP unit cell [43,56,155]. A Chol:Cer[EOS] ratio of 1:2 has been identified as the minimum Chol content required for LPP formation, allowing for a certain degree of LPP flexibility [56,155]. The precise location of Chol in the LPP is unknown; nevertheless, it is unlikely to be predominantly situated in the middle lipid layer of the LPP unit cell [158]. Therefore, Chol potentially bridges the gap between the inner headgroup and the chains of the sphingoid base from Cers and FFAs extending from the unit cell border by locating them in the outer layer of the LPP and contributing to the increased density in the structure [155,159]. Although variations in the FFA content appear to exert a less critical influence on LPP formation compared to the Chol content, a specific range of FFA chain lengths remains essential to promote Chol solubilisation [124]. This aspect was previously mentioned, in which FFA decreased the Chol-phase separation. Moreover, for a proper lipid packing, it is crucial to maintain an optimal chain length variation within the lipid mixture, whether in the FFAs or the Cer fractions [49]. Assuming the Cer[EOS] headgroup is at the unit cell boundary, its C30 acyl chain extends towards the unit cell centre, terminating at the ester bond linking the linoleate [159]. Furthermore, McIntosh et al. demonstrated that Chol may be preferentially located at the outer sides of the LPP cell, which is composed of two bilayers with Chol asymmetrically distributed in each bilayer [57]. The hydrophobic part of Chol aligns closely with the saturated acyl chain of Cers, resulting in strong van der Waals interactions [57,159]. Assuming a perpendicular orientation to the basal plane of Cer[EOS], its ester group must be located very close to the Chol headgroup [159]. While the presence of the unsaturated linoleate moiety in the hydrophilic headgroup region is uncommon, it can be supported by Chol stabilisation. This support may arise from favouring hydrogen bonding with the carbonyl group, entropic stabilisation due to the high conformational disordering of the linoleate, and the limited headgroup hydrophilicity compared to phospholipid bilayers because the SC lipid matrix typically contains only one to two bound water molecules per lipid molecule [152,159], supported by the limited sensitivity of LPP to hydration levels [56,58]. Notably, variations in the headgroup assembly of Cers did not appear to be a prerequisite for LPP formation [155].

In a composition comprising Cer[EOS]:Cer[AP]:Chol (33:22:45 wt%), a lamellar phase with repeated distances resembling SPP was formed, accompanied by a Chol phase separated. This suggests that Cer[EOS] can also be accommodated within the SPP phase, possibly by spanning a layer and extending its acyl chains into neighbouring layers [59]. Interestingly, the addition of PA did not lead to LPP formation, probably due to differences in the chain lengths between short-chained PA and long-chained Cer[EOS], which hindered proper lipid mixing [59]. Similar findings were obtained when PA was replaced with longer-chained BA, TA, or CA [60]. Hence, it is important to acknowledge that the presence of Cer[EOS] is not the only prerequisite for LPP formation, and other additional parameters must be balanced for LPP formation with proper lipid miscibility.

### 3.2. The Short Periodicity Phase (SPP)

**Figure 4:**
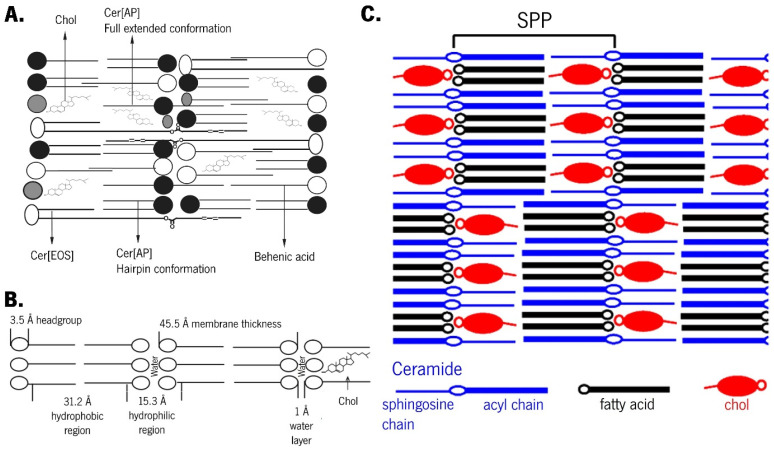
Schematic illustrations of a short periodicity phase (SPP) structure, highlighting (**A**) the Cer[EOS] accommodation on SPP. Adapted with permission from [60]. Copyright 2009, Elsevier. (**B**) The reinforcement model. Adapted with permission from [61]. Copyright 2005, Springer Nature. (**C**) The asymmetric lamellae with alternating directions. Adapted with permission from [126]. Copyright 2014, American Chemical Society.

The suggestion that Cer[EOS] can be accommodated within the SPP phase [59,60] (Figure 4A), along with other evidence of Cers adopting different conformations when present in either LPP or SPP, has led to the armature reinforcement model.

This model is an extension of the sandwich model but specifically applies to the SPP [34]. Cers, as double-chain amphiphiles, can adopt either a hairpin conformation with both chains pointing in the same direction or a fully extended conformation with chains pointing in opposite directions [126]. The adhesion effect promoted by an extremely small intermembrane space is critical for the sandwich model, as the conformational features of Cers are associated with small-sized intermembrane spaces [162]. While several models for the orientation of the hydrocarbon chains have been proposed, the key distinction between the sandwich and armature reinforcement models lies in the postulation of a limited number of Cers in a fully extended conformation while the remaining Cers adopt a hairpin conformation [62,165]. Therefore, the fully extended conformation of Cers serves to strengthen the structural framework [162], which is symbolised by the headgroups linking the opposing headgroup regions of two adjacent leaflets.

Cer[AP] typically arranges symmetrically between the two leaflets of the bilayer, with its polar group serving as a bridge between the leaflets. The polar groups of opposite bilayers form the interface between two membrane leaflets, which is described as the polar headgroup adhesion effect [61]. The almost nonexistent intermembrane space is a consequence of this effect and imposes that the absence of a water layer between subunits creates optimal conditions for the permeation of Cer[EOS] between two layers [61]. Due to the strong lateral hydrogen bonding provided by Cer[AP], Cer[EOS] is obligated to arrange itself in the highly stable SPP [45,59]. This Cer[AP] imposition is also true for various long-chain FFAs arranged within SPP through chain interdigitation in the centre of the membrane or the formation of FFA-rich phases [59]. A model system containing Cer[NP]:Cer[AP] has been found to form lamellar structures with a thickness resembling the native SPP, wherein the long tails overlap within the lamellar centre, as observed in native SC. Interestingly, differences were reported depending on the dominant Cer present in the mixture. A higher content of Cer[AP] resulted in acyl chains with a more tilted packing, reducing their overlap within the midplane but having no effect on the lamellar phase. In contrast, a predominance of native-like Cer[NP] led to straight chains with a broad overlapping region in the lamellar midplane [147]. When used as the sole Cer in the SC model system, a Cer[AP] with 18 carbon atoms induced the formation of SPP [107]. Phase separation imposed by different Cer[AP] conformations was observed in a mixture of Cer[AP]:Chol:SA at a 1:0.7:1 molar ratio, which persisted even after the heating/cooling cycle [110,111]. Although a fully extended conformation of Cer[AP] is highly stable and energetically preferred [105,110,111], the introduction of large amounts of water and/or energy (e.g., heat) can result in a chain-flip of Cer[AP] molecules [61]. This supports the reinforcement model (Figure 4B) [63,162]: after hydration in excess water, the short chains of Cer[AP] undergo a chain-flip transition from a fully extended to hairpin conformation, resulting in increased intermembrane space, allowing water to enter and the disappearance of the narrow contact between adjacent layers [63]. The lipid mixtures of Cer[AP]:Chol:PA:ChS (55:25:15:5 wt%) have been characterised [61,64,65,105] and employed to test the permeation enhancement ability of various chemical compounds [66,104,106]. While vesicular preparation demonstrated phase separation [66,105], which was generally attributed to hairpin and fully extended conformations [166], when the mixture is prepared in multiple lipid bilayer stacks, no phase separation occurs, and a high degree of lamellar order is described [61,104,106]. The only phase in this system is assigned to a fully extended conformation of Cer[AP], which is known to promote an extremely strong intermembrane attraction, resulting in closer neighbouring bilayers, dense contact, and reduced water diffusion in the lateral direction [61] (Figure 4C).

The acyl chains of Cers and FFA cause interdigitation in the SPP, with Chol compensating for the long FFA chains that extend beyond the bilayer’s core [126,158]. Cer[NS] and FFA C24 acyl chains exhibited distinct assemblies within the cores of the LPP and SPP [57,152]. In a mixture with Cer, Chol, and FFA, the long Cer[NS16] or very long-chained Cer[NS24] exhibited a fully extended conformation, which persisted with or without ChS and under hydration [126]. Models containing Cer[NS16] showed lower ordering and miscibility of this Cer with tested FFAs when compared to its C24 homologue. Although the results suggest either a hairpin or extended conformation of the used Cer with a random distribution of its symmetric chains, the hairpin conformation would result in a cross-sectional area very close to that of Chol. This proximity may facilitate miscibility and explain the higher sensitivity of Cer[NS16]-containing mixtures compared to Cer[NS24]-containing mixtures. Moreover, although LA mixed well with the Cer[NS24] in a SPP lamellar phase, some phase separation of Chol occurred, either with or without ChS [126]. These results contradict an asymmetric assembly of SPP in which Cer and FFA chains were partially interdigitated with Chol symmetrically located in both leaflets of the bilayer, slightly inward from the SPP unit boundary [152,153]. Therefore, Školová et al. purposed an explanation for these observations based on two hypotheses: (1) the use of a heterogeneous mixture of Cer that would affect lipid mixing; (2) it can be consistent at a macroscopic scale if the asymmetric assembly with extended Cer is considered, wherein Chol is associated with its sphingosine chain and FFA with its acyl chain, alternating in orientation rather than aligning in the same direction [126].

### 3.3. Cholesterol Influence on Stratum Corneum Lipid Assembly

**Figure 5:**
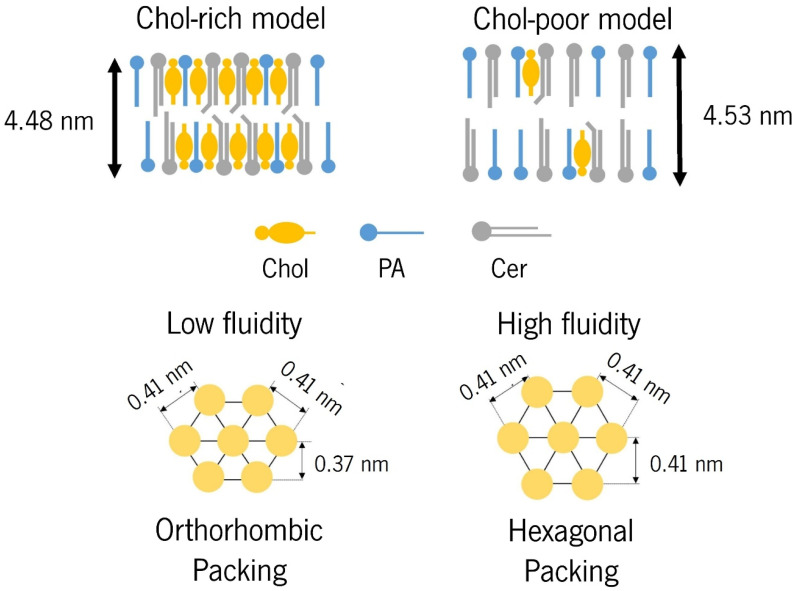
Schematic diagram of the cholesterol (Chol) influence on intercellular lipid matrix organisation. Adapted with permission from [125]. Copyright 2022, Elsevier.

The influence of Chol on lipid assembly was explored in a SC membrane model comprising Cer[EOS]:Cer[NS]:Cer[NP24]:Cer[AS]:Cer[NP16]:Cer[AP] as the Cer mixture [155]. The study revealed that, in addition to increasing lipid density, Chol played a crucial role in the formation of both LPP and orthorhombic lateral packing in SPP [155]. The proportion of lipids engaging in orthorhombical packing increased gradually with the increasing Chol content. This phenomenon could be attributed to the positioning of Chol within the gaps between the very long-chained FFAs, resulting in tighter orthorhombic packing (Figure 5) [125]. Actually, an inverse relationship could be observed between the Chol content and the occurrence of hexagonal lipid packing [125]. A maximum Chol level of 50% relative to the level of FFA was identified, as further increments led to the absence of a liquid-ordered phase [155]. Although Chol exhibited similar effects on the lateral packing of both LPP and SPP, some differences were observed: a larger fraction of lipid adopted orthorhombic packing in LPP compared to SPP, suggesting a distinct distribution of Chol in the LPP and SPP [155]. Additionally, a linear trend of decreasing repeated distances with the increasing Chol content in the membrane after heating/cooling cycles was noticed. The authors proposed two possible interpretations, neither of which precludes their coexistence: Chol increased the tilt angle with respect to the base plane of the membrane and/or Chol reduced the order of the hydrocarbon chains of other membrane components [67,111].

Where various studies have highlighted Chol as a significant factor influencing the miscibility of Cers and FFA, with substantial impacts in the thermotropic behaviour of SC lipid mixtures [68,155], phase interdigitation of Chol-rich crystalline domains has been frequently reported not only in SC lipid model systems [67,111] but also in native SC [167]. Pursuing a deeper comprehension of the Chol influence on the SC lipid membrane structure, Zbytovskáa et al. conducted a study combining different ratios of Cer[AP]:PA:Chol:ChS [67]. Their findings revealed that, for all the tested ratios, the fluidising effect of Chol was evident when the system was below the main phase transition temperature (T_m_). Conversely, when the lipid system was above the T_m_, Chol increased the chain order [67].

### 3.4. Phase Separation and Promoters of Miscibility on Stratum Corneum Lipid Models

Pure Cer[NP] is characterised by its high melting temperature, forming crystalline orthorhombic structures [69,116,133]. Both Cer[NS] and Cer[NP] occupy the same location within the LPP unit, adopting predominantly an extended conformation with the (phyto)sphingosine and acyl chains on either side of the headgroup [151,168]. This linear conformation benefits the skin’s barrier function by reducing the cross-sectional area per lipid molecule compared to the hairpin conformation, which enables a tighter packing of hydrocarbon chains [168]. Additionally, it enhances the connection between adjacent lipid layers, reducing permeability in the SC model system and discouraging lipid lamellae swelling during hydration [139]. Indeed, at room temperature, SC lipid mixtures typically exhibit a significant portion of their lipids in a solid crystalline phase [55]. The presence of phase-separated domains of Cers and FFAs arranged in an orthorhombic chain packing contributes to the low permeability of skin [69,70,71,72]. Subsequently, further homogenisation and the formation of a liquid crystalline phase occur at high temperatures [68,70,73]. Ordered-disordered phase transition has also been observed at temperatures similar to those found in native SC lipids [55,70,72,74].

In a model using _BB_Cer[NP] in combination with Chol and PA at variable ratios, a common trend is observed: increased miscibility with the rising temperature. However, a high Chol content results in a delayed attainment of complete miscibility (from the 40–50 °C range to 75 °C) [73]. In Cer[NS] mixed with Chol or SA, Chol exhibited good miscibility with Cer at physiological temperatures and across a wide range of temperatures. In contrast, systems containing SA tend to phase separate into a SA-rich phase, achieving only moderate miscibility at high temperatures [68]. The kinetics of orthorhombic domain formation in SC lipid models were monitored via infrared spectroscopy by Mendelsohn et al. [122]. Conformational ordering and orthorhombic packing formation within the Cer[NS] chains occurred on a similar timescale as Cer headgroup region changes. In contrast, the formation of hydrogen-bonded dimers of SA started with a time lag of 3 to 4 h. The formation of FFA domains, along with conformational ordering of the lipid chains, likely required physical separation between hydrophobic regions, enabling their chains to segregate into orthorhombic domains [122]. The phenomenon of increased homogeneity with the temperature is contingent on several factors. Swelling in the LPP can be induced by elevated pH values or a high content of ChS, which promotes fluid phase formation due to its negatively charged sulphate group [57]. The sulphate group increases the molecular area per lipid, reduces the lipid packing density, enhances lipid miscibility, and strengthens its ability to form more hydrogen bonds with water compared to the OH group of Chol [51,108].

In the case of Cer[NP]:Chol:SA (55:25:20 *w*/*w*), phase separation occurs, with one phase exclusively composed of hairpin Cer[NP] molecules and the other phase consisting of a mixture of all components from the system, including fully extended Cer[NP] molecules that bridge adjacent bilayers [42]. In the hairpin conformation, the polar headgroup of Cer[NP18] is positioned between the two acyl chains, which are oriented in opposite directions [42]. Interestingly, this latter phase did not respond to applied contrast variation, as the headgroup of Cer[NP24] effectively shields against intrusion by water or deuterated water through the outstretched acyl chains and inter- and intramolecular hydrogen networks [42,157]. In equimolar Cer[NP]:Chol:LA, two phases coexist, with one phase being rich in fully extended Cer[NP24] and the other of reduced thickness, suggesting a distinct membrane structure and/or composition, possibly involving intercalated and/or tilted chains [116]. In systems involving Cer[NP] alone or combined with Cer[NS] or Cer[AP]:[NS] mixed with Chol:SA+ChS at a molar ratio of 1:1:1 + 5 wt%, three different phases were identified, corresponding to a phase rich in Cer[NP] with a minor incorporation of Chol and SA, a phase with high miscibility degree among all the components, and another phase resembling a pure SA phase with a minor presence of other components [133].

Although increasing the hydration or temperature induced phase separation in Cer[NS] mixtures, the demixing extent was highly dependent on the FFA chain length [120]. The presence of shorter FFAs reduced the temperature of the orthorhombic to hexagonal packing transition [120]. In equimolar mixtures of Cers, Chol, and FFA containing Cer[NS], Cer[NP], Cer[NP-18:1], or Cer[AS], the lipid assembly was dependent on the employed Cer [123]. Differences in the hydrocarbon chains were also investigated by comparing Cer[NP]- and Cer[NP-18:1]-based mixtures. Phase segregation was observed in the Cer[NP]-based system, whereas the use of Cer[NP18] promoted the formation of a more homogeneous phase [123]. The formation of solid crystalline phases results in ordering all the methylene segments into an all-*trans* configuration to maximise the van der Waals interaction, and maybe the double bond present at the middle of hydrocarbon chain leads to a strong reduction in the ability of Cer[NP-18:1] to form solid crystalline phases [123]. Depending on the used Cer, variations in the mixing properties with PA were noticed, including the coexistence of orthorhombic and hexagonal packing, along with the phase segregation of PA enriched with Chol from the phases of Cer[NP] with PA [123]. However, when SA is used instead of PA, both lipids adopt hexagonal chain packing, making mixing more likely [75]. The phase separation observed between FFAs and Cer has been attributed to a hydrophobic mismatch between their chain lengths [67,133,146]. The effect of FFA on LPP was notably dependent on their chain length (ranging from C16 to C28) [146]. Longer FFAs increased the repeat distances of the lamellar phase, whereas shorter FFAs had the opposite effect. Notwithstanding, whereas the FFA chain length affected the lateral packing, the orthorhombic to hexagonal packing occuring in phase transition was not affected. When shorter FFAs were neighbouring longer Cer C24 acyl chains, it caused hydrophobic mismatching and elastic stress on the lipid lamellae. When the amount of stress exceeds the level that the lamellae can accommodate, a lipid reassembly is required to relieve it, resulting in phase separation or the formation of more energetically favourable structures [156]. Although the presence of FFAs with varying acyl chain lengths is regarded as a promoter of lipid miscibility [52], it is only evidenced when matching FFA chain lengths and Cer acyl chains occur [120]. For example, a mixture containing six FFAs with different chain lengths in a SC model system inhibited the separation of individual FFAs from the multilamellar structure, likely due to a partial interdigitation that resulted in free spaces within the system, which were filled by FFA through tilted chains towards free volume minimisation inside the system [108]. Contrastingly, Engstrom et al. observed a tendency for polar lipids to segregate in the crystalline state, even when the lipids belonged to the same family [76]. This phase separation is attributed to packing constraints imposed by differences in chain length, particularly when the disparity in hydrocarbon chain length exceeds four carbon atoms [76]. Furthermore, there is a general propensity for the presence of very long-chained FFA to favour the mixing of lipid components, as reported in other studies involving SC lipid systems containing Cer[NS24] [120,127]. Paz Ramos et al. found that Cer[NS] combined with C24 FFAs led to mixtures with significantly greater homogeneity than those incorporating chains containing C16 or C20. Remarkably, after a week of incubation, the C24 system preserved its homogeneity, whereas systems containing FFA with C20 and C16 showed notable variations in local composition over time [127]. In the context of phase separation, it appears that the intermolecular interactions are the primary driving force, which suggests that very long acyl chains contribute to a relative increase in van der Waals interactions, thereby promoting lipid mixing in the SC lipid matrix [127]. Notwithstanding, other studies have proposed that chain heterogeneity might play a more crucial role in enhancing lipid miscibility compared to hydrophobic chain matching [77]. This was exemplified by cases where even with optimal hydrophobic matching between components, such as in the Cer[NS16]:Chol:FFA C16 mixture, extensive phase separation occurred [77]. It is worth noting that FFAs do not form hydrogen bonds with themselves due to the unavailability of undissociated hydrogen atoms in their headgroups; instead, their oxygen atoms are available for hydrogen bonding with other lipids [135]. Considering that FFA preferentially forms hydrogen bonds with dominant Cer molecules [135], it becomes apparent that the polarity of the headgroup also influences the miscibility characteristics of SC lipid mixtures. Indeed, the influence of the headgroup can outweigh the influence of the chain length in terms of the membrane assembly [45,59,78]. In experiments involving monolayers composed of variable combinations of _pig_Cer, _BB_Cer, Cer[NP16], or Cer[NP24], along with Chol and PA (C16) or LA (C24), phase separation into distinct lipid domains was observed. For instance, short-chain Cer[NP16] mixed with Chol and PA, while long-chain Cer[NP24] formed domains independently without mixing with LA [78]. Furthermore, the internal nanostructure of the Cer[AP] systems was not linearly affected by an increase in the FFA chain length in the system (from C18 to C26) [45]. Initially, the increase in FFA chain length led to a decrease in the membrane repeated distance through a partial interdigitation of the FFAs chains. This interdigitation resulted from the tendency of FFAs to ‘fit’ into the membrane size created by Cer[AP]. The authors explained that the formation of FFA-rich phases in the presence of longer-chained FFAs with C24 and C26 chains was due to the lower solubility of long-chained FFA in the Cer[AP]-based SC system [45]. This propensity of longer-chain FFA to form separated FFA-rich phases in Cer[AP]-based SC systems, even in presence of Cer[EOS], was also reported by Kessner et al. [59]. Therefore, interactions mediated by Cer[AP] molecules appear to be the main forces determining the stability and dictating the main phase of the system [45,59].

Consequently, the presence of FFA exerts a notable influence on the packing of Cer molecules. When Cer[NS]:Cer[AP] at 2:1 and 1:2 molar ratios are mixed with Chol and LA, a single lamellar phase is formed with the formation of FFA- and Cer-enriched nano-sized domains, yet without complete phase separation. Moreover, Chol was uniformly distributed throughout the system [140]. This reinforces the limited miscibility of Cer[NS] with other lipids [68,77,120,126]. Interestingly, a smaller amount of Cer[AP] exhibited good miscibility with a higher quantity of Cer[NS], but the reverse scenario was not observed. In these systems, the lipid chains exhibited a slight tilt, and an assembly of Cer molecules with long overhanging tail ends of Cer C24 acyl chains and the long LA C24 chains overlapping in the lamellar middle was proposed. This was counteracted by the shorter C18 chain of the sphingoid base and Chol, allowing the opposing longer chains to fill these gaps [140]. On other hand, Chol is described as ‘line active’, since it tends to promote the miscibility of lipids [76,78], which is possible through the maximisation of hydrophobic interactions with alkyl chains from Cers and FFA [64].

### 3.5. Ceramide Headgroup Influence on Stratum Corneum Assembly

The effects of the Cer headgroup on the assembly of SC membrane lipids have been extensively explored [42,70,122,123,133,149,160]. Cer headgroups play a crucial role in governing the behaviour of LPP and SPP, impacting parameters like packing density, lipid miscibility, and hydrogen bonding strength [75,169]. Engelbrecht et al. conducted a study focusing on the replacement of Cer[AP] with Cer[NP] within SC lipid membranes containing SA and Chol. This substitution, which involved the removal of a single OH group from the Cer molecule, led to drastic structural alterations [42]. Although both were important in conferring high lamellar order to SC models, the Cer[AP]-containing system exhibited a more homogeneous mixture with a lower lamellar order than the one containing Cer[NP], which showed the coexistence of two lamellar phases [42,75]. Both Cers induced the formation of a solid crystalline phase with tightly packed and aligned hydrocarbon chains. Cer[AS]m, which has an extra OH group and can adopt different chain conformations, can also arrange its chains in a hexagonal lattice [123]. Moreover, the absence of an OH group in the headgroup region appeared to prevent hydration or swelling of the SC model membrane, possibly due to strong intra- and intermolecular headgroup interactions [42]. Badhe et al. demonstrated that differences between Cer[AP] and Cer[NP] could be attributed to variations in the hydrogen bonding dynamics. In a study with both Cer[AP] and Cer[NP] at 2:1 or 1:2 ratios, they found that the dynamics of hydrogen bonding with FFA and Chol changed significantly depending on the concentration of Cers [135]. Surprisingly, even though the additional OH group in Cer[AP] should facilitate greater hydrogen bonding, a dominant presence of Cer[AP] resulted in less ordered packing. This suggests that hydrogen bonding and the localisation of OH groups both play important roles in determining the packing order [133,135]. The additional OH group in Cer[AP] caused a tilt in the hydrocarbon chains, affecting their mobility and leading to a disrupted packing [135]. Packing defects due to the extra OH group were also reported, with a Cer[NP] system exhibiting disturbed packing compared to a Cer[NS] [160]. The extra OH group generated a steric conflict, reducing the lateral packing and, consequently, shifted the phase transition of the liquid crystalline phase to lower temperatures [160]. The impact of a single OH group on phase separation kinetics and subsequent barrier function was also observed when Cer[NS] was replaced by Cer[AS]. The extra OH group in Cer[AS] altered the propensity and quantity of the hydrogen bonds formed by both the Cers and FFA headgroups [122]. The removal of an OH group and the introduction of a trans-double bond in position 4 of an C18 Cer[NP] increased the solid crystalline phase compared to the C24 variant [160,170]. Longer C24 chains compensated for the packing defect induced by the additional OH group of Cer[NP], likely due to more methylene segments contributing to van der Waals interactions, compensating for the entropic losses caused by steric conflicts at the headgroup region [160]. Contrastingly, decreasing the chain length to C16 led to less ordered phases [160]. Another study demonstrated that, while adding Cer[NH] with three OH groups or Cer[AP] with four OH groups to a ternary mixture containing Cer[NS] did not interfere with the formation of the solid crystalline phase, the incorporation of tri- or tretrahydroxylated Cers resulted in stronger forces holding the lipid system together [142]. The most significant difference between Cer[AP] and Cer[NH] is the ability of the latter to promote the formation of a lamellar phase close to LPP, which appears to be related to the presence of OH at position 6 of the sphingoid backbone [142]. In another approach, the two stereometric forms of Cer[AP]: *D*- and *L*-isomers [166] were incorporated into ternary or quaternary mixtures [137]. While ternary mixtures containing only *D*-isomer as Cer exhibited crystalline-like behaviour, quaternary mixtures incorporating Cer[NP] exhibited a more SC lipid matrix-like behaviour [137]. This mixture was arranged with mostly straight chains and overlapping long chains in the lamellar midplane [137]. In the case of the *L*-isomer, no lamellar phase was found when it was the only Cer in the mixture, and when combined with Cer[NP], a highly crystalline-like behaviour was reported, classifying it as not safe [137]. These distinctions can be attributed to the reported hairpin conformation associated with the *L*-enantiomer of Cer[AP], whereas the *D*-enantiomer is associated with the non-tilted form, which is eventually associated with longer repeat distances [110]. Sphingomyelin (SPM) is a precursor of skin Cers, and its presence in a mixture with Chol and PA instead of Cer demonstrated how different headgroup structures have a significant influence on the SC structure [70]. While the SPM-based mixture formed a homogeneous liquid ordered mixture, replacing it with a Cer introduced a rich polymorphism into the structure. Additionally, while the Cer-based system showed a highly ordered structure with Cer and FFA residing in an orthorhombic subcell at low temperatures, a homogenisation with the formation of a fluid phase was reported at high temperatures [70]. Cer[EOP] and Cer[EOS], which differ in terms of the presence of an additional OH group and 4,5-desaturation, showed distinct abilities to promote the formation of LPP, with Cer[EOS] being more efficient in this regard [49].

Nonetheless, there are also examples of lamellar phases that are adaptable to differences in lipid headgroup assembly. While Cer[NP] and Cer[NS] occupy similar positions in the LPP unit, water permeation can influence their crystalline phases due to an increased opening angle of the hairpin conformation with higher hydration levels [123,137,151]. A 2–3 °C shift in the orthorhombic to hexagonal transition temperature was associated with increased hydration conditions, which was attributed to weakened hydrogen bonds between opposing headgroups of lipid layers induced by the incorporation of small amounts of water into neighbouring layers of headgroups [120]. The optimal hydrogen bonding of the hydrated phytosphingosine headgroup was found to be incompatible with orthorhombic chain packing [165]. This change in orthorhombic packing induced by Cer[NP18] was not related to the missing *trans* double bond in the phytosphingosine backbone [160]. The extent of orthorhombic chain packing of Cers depends on factors such as their chemical structure, temperature, and hydration degree [123]. Higher-phase transition temperatures are associated with stronger interactions in the headgroup region, primarily between OH groups and the amide group. Phytosphingosine-based Cers exhibit extremely low amide I wavenumbers, indicating a strong involvement of these amide groups in a tight hydrogen bond network. Conversely, sphingosine-based mixtures show higher amide I frequencies, suggesting weaker hydrogen bonding interactions between headgroups [123]. Monolayer measurements have revealed that the headgroup area of phytosphingosines is larger when compared to sphingosine Cers, and a more open polar interface allows for a stronger hydrogen bond network [123].

### 3.6. Main Considerations for Stratum Corneum Barrier Function

Lipids in the LPP are arranged in two layers sandwiching a narrow central lipid layer containing a subpopulation of fluid lipids. In SC lipid systems, the formation of the LPP is dependent on achieving an optimal fraction of lipids that form a liquid-like phase. When this fraction deviates from the optimal range, either by being too low or too high, it leads to an increase in SPP formation at the expense of LPP formation [50]. Specifically, the linoleic acid moiety of Cer[EOS] is found in this central layer [50].

Fluid phases exhibit significantly higher permeability compared to crystalline phases. Given that the fluid phase is situated in the central layer of the LPP, permeation parallel to the basal plane of lamellae is faster than permeation perpendicular to the lamellae (across headgroup regions) [50]. An increased fraction of unsaturated acyl chains, particularly those linked to the long base, enhances the fluidity and, consequently, permeability. Membrane permeability is directly affected by the relative complexity of the SC lipid system used [133]. Maintaining a balanced relationship between the three key properties—orthorhombic packing, hydrogen bonding, and miscibility—is critical for establishing an effective barrier.

To summarise, the most critical characteristics of SC lipid systems include (1) a wide distribution of acyl chain lengths in Cers; (2) the coexistence of Cers based either on phytosphingosine, sphingosine, or 6-hydroxysphingosine bases in a single mixture; (3) the presence of acylCers; and (4) a range of FFA chain lengths varying between C16 and C26. These characteristics play a pivotal role in influencing lipid phase behaviour, with acylCers being crucial for LPP formation and the need for a broad distribution of FFA chain lengths to form both LPP and SPP without additional coexisting phases rich in FFA or Cers, replicating the lipid assembly found in human SC.

## 4. *Stratum Corneum* Lipid Models as Surrogates for Permeation Studies

In recent years, significant effort has been directed toward the development of skin surrogates that better mimick the complexity of the SC ILM. This endeavour arises from both the simplicity and intricacy of existing SC lipid model systems, along with the limitation associated with the current skin surrogates. Therefore, Table 2 provides a summary of recent developments in innovative SC surrogates (SCS) based on SC lipid models.

**Figure 6:**
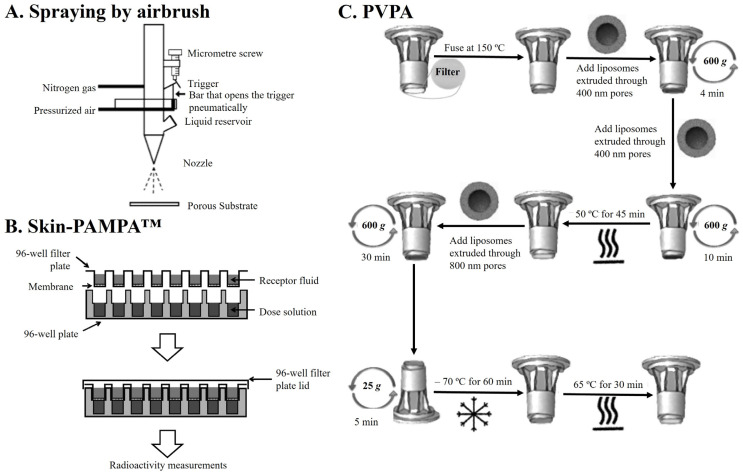
Schematic representation of each approach described to obtain *Stratum Corneum* lipid mixture-based surrogates for in vitro permeation evaluation. (**A**) Spraying by airbrush. Adapted with permission from [58]. Copyright 2006, Elsevier. (**B**) Skin-Parallel Artificial Membrane Permeability Assay (Skin-PAMPA^TM^). Adapted with permission from [197]. Copyright 2013, Elsevier. (**C**) Phospholipid Vesicle-based Permeation Assay (PVPA). Adapted with permission from [189]. Copyright 2013, Wiley.

From a general point of view, a SCS can be described as a synthetic model of SC ILM that consists of depositing an appropriate mixture of lipids mimicking a SC ILM composition on a porous substrate. This porous substrate covered by the lipid mixture intends to be mounted in a typical diffusion cell between donor and acceptor compartments (Figure 6A). What differentiates the type of SCS is the approach followed to cover the filter membranes with lipids. Therefore, the approaches can be divided into spraying by airbrush (Figure 6A), the Skin-PAMPA^TM^ (Figure 6B), and the Phospholipid Vesicle-based Permeation Assay (PVPA) (Figure 6C).

### 4.1. Spraying by Airbrush

The deposition of the SC lipid mixture onto a porous substrate via the spraying method entails a series of steps that have been optimised through time. Initially, lipids are dissolved in a hexane:96% ethanol (2:1 *v*/*v*) solution to a final desired concentration of 4.5 mg∙mL^−1^. Subsequently, this solution is sprayed onto the substrate under a nitrogen flow, typically employing a Linomat system with an extended y-arm (Figure 6A). Following that, an incubation period is employed, which can vary in temperature (from 70 to 90 °C, depending on the Tm of the used mixture) and duration (from 10 to 30 min), culminating in a cooling step to room temperature that can last 30 min, 3 h, or overnight.

In studies comparing three different methods for lipid airbrushing—manual, using a rotor, or the Linomat—the Linomat apparatus was found to be the most suitable for preparing SCS due to its efficiency and the uniformity of the membrane thickness compared to the other two methods. Although using benzoic acid as model compound, the steady-state flux was similar for the three SCS, and the values were very similar to human SC, so the authors selected the Linomat as the most appropriate apparatus for preparing the SCS [185]. Furthermore, the most critical factors in the spraying protocol to ensure the suitability of the SCS for permeability tests, in which it is imperative to obtain a densely packed and uniform layer, were investigated [58]. The research findings suggest that these critical factors include (i) the total lipid concentration; (ii) the composition of the organic solvent mixture; and (iii) the spraying equipment parameters, like the distance between the airbrush and the filter, the nitrogen pressure during both spraying and drying stages, and the amount of lipid reaching the porous substrate [58]. Deviation in the process conditions can lead in suboptimal outcomes, such as organic solvent evaporation occurring from the inside to the periphery and potential overspray of lipid solution onto the periphery of the filters, which typically occurs when nitrogen pressure is too high or the evaporation rates for the lipid solution are inadequate. During solvent evaporation, the lipid concentration increases, leading to lipid crystallisation based on their solubility. In diffusion experiments, non-hydrated lipid membranes must maintain their integrity when exposed to donor and acceptor solutions for an extended duration. Preliminary studies indicated that the SCS remained intact for at least 20 h during a typical diffusion experiment [58]. The lipid membranes cooled to room temperature before hydration exhibited minimal pore formation, with no water pools within the membranes at room temperature [58].

The use of this approach is prevalent in the scientific literature, and it is used not exclusively for permeation experiments but also for the biophysical and dynamical characterisation of SC permeability. An equimolar mixture of Cer[NS]:Chol:PA airbrushed in polycarbonate membranes formed a gel-phase structure capable of maintaining robustness even at high temperatures [175]. The permeability of caffeine (CAF) and benzoic acid in this SCS was comparable to that of real SC. However, there were variations in the membrane thickness (ranging from 16 to 26 μm) due to some sprayed droplets not coalescing into the continuous layer [175]. SCS composed solely of phytosphingosine Cers (Cer[NP] and Cer[AP]) demonstrated permeability characteristics similar to human skin when theophylline (TH) and indomethacin (IND) were used as model compounds [179]. However, the steady-state time for both permeants in SC membranes was shorter than in human skin. Variable ratios of the Cers content were tested, and while Cer[AP] showed the highest TH permeability, there was no significant difference from the TH and IND flux in porcine skin [179]. The influence of sphingosine- and phytosphingosine-based Cers on SCS permeability was compared. The steady-state flux of ethyl-PABA was significantly lower in phytosphingosine-based SCS compared to their sphingosine counterparts. There was no significant difference in the steady-state flux of ethyl-PABA between SCS with non-hydroxy fatty acid-based Cers and their α-hydroxy fatty acid counterparts [169]. Kovacik et al. investigated the effects of Cer α-hydroxylation and stereochemistry on the barrier properties. Both saturated sphingosine double bonds of Cer[NdS] and Cer[NP] and α-hydroxylation increased the water loss compared to the control Cer[NS], except for Cer(R)-[AdS], where water loss did not change. TH, a small molecule with balanced lipophilicity, showed decreased permeability upon Cer α-hydroxylation, with a significant difference between Cer(R)-[AdS] and Cer(S)-[AdS] diastereomers. IND, being larger and more lipophilic, exhibited similar permeability across α-hydroxylated Cers, except for Cer(R)-[AdS] and Cer(S)-[AdS] diastereomers, which showed twice the permeability of Cer[NdS] [37]. Furthermore, Kovácik et al. synthesised several unnatural Cer[NS]: 1-deoxy-, 3-deoxy-, and N-Me- and combined them with LA, Chol, and ChS for permeability testing [36]. Modifications at the polar headgroup had a significant effect on the SC model properties. The presence of an OH group at the C1 position was crucial for a proper lipid mixture, as the 1-deoxy-Cer[NS]-based system exhibited significantly higher permeability compared to physiological Cer[NS]. The N-methylation of Cer[NS] increased the water loss threefold and TH and IND permeability tenfold compared to the control [36]. Moreover, the influence of increasing the Cer[EOS] content in SCS barrier function was investigated [183]. Increasing the Cer[EOS] content from 10 to 30% of the total Cer fraction did not significantly affect the ethyl-PABA permeability. However, a further increase to 50, 70, and 90% of the Cer[EOS] content resulted in enhanced permeability, accompanied by increased water loss at higher Cer[EOS] contents (70 and 90%), attributed to the formation of a liquid-like phase.

The SCS were also examined for their utility in testing permeation enhancers, particularly those affecting the SC lipid matrix, such as Azone^®^ and L-Pro2 [183]. Groen et al. examined the suitability of a SCS to mimick human SC by studying the interactions between the SC lipid system and three models from a class of chemicals frequently used as fragrance raw materials: γ-undecalactone, dodecyl acetate, and diethyl 1,4-cyclohexanedicarboxylate [174]. Between the several advantages, including the possibility to investigate the hydrogen bonding between headgroups, one limitation was assigned to SCS models over SC by tape-stripping: the impossibility so far to determine the in-depth profile of compounds [174].

### 4.2. Skin-Parallel Artificial Membrane Permeability Assay (Skin-PAMPA^TM^)

Originally, the PAMPA method was developed for the rapid assessment of the passive membrane transport of molecules. Due to its potential cost-effectiveness and high-throughput capabilities, PAMPA has been widely adopted in the pharmaceutical industry [80]. The PAMPA method involves a 96-well plate with a filter that separates two compartments: the donor compartment containing a buffer solution of the compound under investigation and the acceptor compartment containing fresh buffer solution (Figure 6B). Over time, adaptations of PAMPA have been made to evaluate the epithelial intestinal barrier and blood–brain barrier (BBB) permeation. Recently, Sinko et al. introduced Skin-PAMPA^TM^. In Skin-PAMPA^TM^, the SC ILM lipid model consists of a lipid mixture containing certramides (instead of Cers), Chol, SA, and silicon oil [80,188]. Certramides, which are analogous of Cers, differ from natural Cers in that they lack unsaturated bonds in the side chain but share structural similarities, such as similar molecular size, hydrogen bond/acceptor capabilities, and high lipophilicity [80,188]. Skin-PAMPA showed a limited correlation with the epidermis, but a stronger correlation was observed with full-thickness skin [188]. When comparing Skin-PAMPA^TM^ with other artificial models and using porcine skin as the reference, permeability data for a selection of drugs solubilised in different vehicles were examined. The results indicated that, among several artificial models, including Strat-M^®^, Skin-PAMPA^TM^ provided the highest correlation by accurately ranking four out of six vehicles [197].

Despite claims of structural similarity between certramides and Cers and good correlations with animal models, the absence of unsaturated bonds and the presence of silicone oil in the mixture may limit the ability of the Skin-PAMPA^TM^ tool to accurately replicate the complex structure of SC ILM. As discussed in the previous section, the composition of the lipid mixture employed has a direct impact on membrane permeability, and the presence of unsaturated acyl chains is critical for the formation of either LPP and liquid-like domains crucial for the proper elasticity of SC lipid models [50,157,159]. Furthermore, analysing the statements taken before as the most critical characteristics of SC lipid systems, Skin-PAMPA^TM^ fails in the coexistence of different bases of Cers in a single mixture and in the presence of acylCers and also fails to guarantee a range of FFA chain lengths varying between C16 and C26.

### 4.3. Phospholipid Vesicle-Based Permeation Assay (PVPA)

The other proposal to obtain SCS is based on a lipid covering barrier created by a tight layer of liposomes on a filter support—PVPA [189,191]. The PVPA model was initially developed to mimick the intestinal barrier based on a membrane filter support with deposited liposomes of a defined composition [198,199]. A schematic representation of the full method to fabricate PVPA membranes is depicted in Figure 6C. The original PVPA model waas prepared by placing the liposomes through centrifugation onto a mixed cellulose filter, followed by solvent evaporation and freeze–thaw cycling to promote liposome fusion, resulting in a tight barrier. This vesicle-based barrier was characterised by unrequired agitation during the experiments, which was explained by the presence of aqueous compartments immobilised within the vesicle’s matrix. Later, a modified version of the PVPA model with phospholipids composed by a SC lipid matrix lipid mixture was designed [189], in which the permeability of different compounds was evaluated and validated by comparison with data obtained from animal skin models and from in silico [189]. The model was then compared to a commercially available reconstructed human skin model—EpiSkin^®^—and the authors reported that the PVPA model was more effective, less expensive, and had longer storage stability than EpiSkin^®^ [191]. Other permeation experiments were conducted based on this approach, including testing the PVPA relevance in preformulation studies and evaluating permeation enhancers [190,192,193,195,196]. Shakel et al. then presented a modified PVPA. The authors sonicated the multilamellar vesicles (MLVs) colloidal dispersion to decrease and homogenise the liposome size towards the adaptation to polycarbonate filter support with 400 nm. Then, the modified PVPA briefly consisted of retaining/passing of a portion of large unilamellar vesicles (LUVs) through a membrane filter with successive centrifugation, followed by freeze–thawing until the pores were filled by the LUVs and a layer on top began to form by the addition of MLVs [195]. While the PVPA from Engesland et al. was stable up to 2 weeks of storage at −70 °C, Moniz et al. showed that their modified PVPA [195] could maintain its integrity and lipid content over 12 weeks when stored at −20 °C, without changes in the calcein (CAL) permeability [196]. In the same investigation, while the modified PVPA model was not affected by the presence of a set of cosolvents that were usually employed as skin permeation enhancers (Tween^®^ 20, Tween^®^ 80, and PEG 400), ethanol at 10% (*v*/*v*) altered the integrity of the barrier [196].

The main disadvantage of the PVPA approach lies in the presence of phosphatidylcholine in the SC mimetic mixture, which does not translate the subclasses characteristically found in SC.

## 5. Commercially Available and Patented Skin Models

The use of skin, whether human or animal, has long been regarded as the gold standard for assessing (trans)dermal permeability. The commercial products like Transderm-Scop^®^, Androderm^®^, and Alora^®^ were excised human skin used as a surrogate for in vivo studies [11,200]. Yet, ethical, technical, and economic challenges have stalled their widespread use. Obtaining human skin poses hurdles due to ethical concerns, high variability, low throughput, and technical challenges, limiting its use. Similarly, obtaining animal skin is time-consuming, technically challenging, and costly, and excised skins do not accurately represent human skin physiology and structure. In addition, both in vivo animal and human studies and ex vivo studies using excised skin models contribute significantly to the carbon footprint of pharmaceutical industries, raising sustainability concerns. The pharmaceutical and industry/research sectors are increasingly inclined to adopt other in vitro testing methods to lessen the reliance on in vivo studies or ex vivo surrogates. This trend is further emphasised by the EU prohibition on animal testing in cosmetic and toxicology evaluations by the directive 76/768/EEC [201], demanding alternative skin permeation testing methods.

In response to this, models equivalent to living skin have been developed by culturing human skin components such as keratinocytes and fibroblasts, and several human skin cell cultures are commercially available as skin models: EpiSkin^TM^ model (Episkin, L’Oréal, Lyon, France), EpiDerm^TM^model (MatTek Corporation, Ashland, MA, USA), SkinEthic^TM^ (Episkin, L’Oréal, Lyon, France), Labskin^TM^ (Labskin, Lyon, France), EpiCS^®^ model (CellSystems, Troisdorf, Germany), Straticell model (Straticell, Les Isnes, Belgium), and Labcyte model (Gamagori, Japan); Full-thickness models such as the StrataTest^®^ model (Stratatech, Madison, WI, USA), Phenion Full-Thickness Skin Model (Phenion, Düsseldorf, Germany), GraftSkin^®^ (Apligraf, Organogenesis, MI, USA), EpiDermFT^®^ (MatTek Corporation, Ashland, MA, USA), and Vitrolife-Skin^TM^ model (Kyoto, Japan) [11,202]. These models have been employed in Franz diffusion cells for permeation assessments or incorporated into microfluidic chips (known as ‘skin-on-chip’). However, they still fail to address several issues related to excised tissue, proving expensive, requiring specialised technical skills, and inadequately replicating human skin permeation. Primarily utilised for skin irritation and toxicity assessments, these cellular models present challenges that surpass the conveniences offered by microfluidic analysis. Despite the potential benefits of microfluidic technology in enhancing screening processes, the integration of skin cell-based models presents obstacles to achieving high-throughput screening. These hindrances include limitations in storage capacity of the cell-based models, lack of reproducibility, technical handling requirements, and elevated screening costs [11].

Finally, non-cellular skin models have been developed to access skin permeation. membranes. Most models are synthetic membranes for transdermal delivery composed of silicone-based membranes such as Silatos^TM^ (LMA, Better by design, London, UK), Silastic^®^ (Dow Corning Corporation, Midland, MI, USA), and multiple layers of polyester sulfone Strat-M^®^ (Merck Millipore, Burlington, MA, USA). While synthetic membranes offer certain advantages over human or animal skins, establishing a definitive correlation with human SC barrier function, especially in finite dose applications, remains a challenge. Additionally, these models, predominantly composed of hydrophobic polymers, exhibit varying permeation efficiencies. While they provide reasonable estimations for hydrophilic compounds, they tend to overestimate the permeation of lipophilic compounds [11].

Recently, there have been advancements in synthetic barriers with compositions that more closely mimick natural skin. For instance, Permeapad^®^ (PHABIOC GmbH, Karlsruhe, Germany, patent WO 2016/078667 A1) [203] features two cellulose membranes with a phospholipid layer in between, while Skin-PAMPA^TM^ (Pion Inc., Billerica, MA, USA), already detailed in the previous section, utilises a model containing a synthetic amide-based compound containing certramide, free fatty acid, and cholesterol. Permeapad^®^ offers a membrane model for use in Franz diffusion cells or as part of the PermeaPad^®^ Plate consisting of two 96-well plates. The upper plate serves as the donor, with an insert plate integrating the membrane model, and the bottom plate acts as the acceptor. The bottom PermeaPad^®^ Plate can be conveniently placed in standard fixtures of 96-well plates from HPLC equipment to quantify permeated compounds. On the other hand, Skin-PAMPA^TM^ is available as the SuperQuick^®^ Skin-PAMPA Kit (Creative bioarray, New York, NY, USA) comprising two 96-well plates, with the upper plate serving as the donor and the bottom plate as the acceptor. The skin model is provided as an organic solution to be added to the donor plate and allowed to dry. Like Permeapad^®^, the bottom plates of Skin-PAMPA^TM^ can be placed in standard fixtures of 96-well plates from HPLC equipment for the quantification of permeated compounds.

Besides Permeapad^®^ and the SuperQuick^®^ Skin-PAMPA Kit, there are no competing high-throughput screening commercially available solutions incorporating cell-free mimetic models of skin. However, some patented devices can be applied for cells-on-chip (US10870823B2 [204], WO2021168511A1 [205], and US2022265176A1 [206]). Few other cell-free skin models have been patented:

CN114577678A—This Chinese national patent presents liposomes hydrated with 10% keratin arranged between two sealed polycarbonate membranes forming a mimetic model of skin. Filed by Nanjing Traditional Chinese Medicine University in 2022, the model offers the advantage of containing keratin, representing a protein component of the skin. However, polycarbonate membranes lack physiological relevance and do not mimick any skin component. There is no associated high-throughput device for a permeation assessment [207].

WO2008011812A1—This international patent describes synthetic phospholipid polymers containing amphipathic regions. Filed by the Shanghai Cancer Institute in 2008, these polymers mimick the dual affinity of the lipid matrix (hydrophilic and hydrophobic portions). However, they do not possess physiological relevance, failing to mimick the lamellar and lateral organisation crucial for the skin’s permeation barrier functions [208].

## 6. Conclusions and Prospects

Over the last 30 years, numerous research groups have contributed to a better understanding of the structure of skin and the mechanisms of its permeation, and due to increasingly sophisticated biophysical techniques, our knowledge on skin permeation has grown markedly. The promising outcomes obtained from the analysis of model drugs and other permeability markers underscore the clear potential of SCS to be employed in a more standardised, less expensive, and more ethical way when investigating drugs or cosmeceuticals, especially those intended for compromised skin conditions, compared to other current models. SCS can potentially reduce the costs and the use of animal testing in the early stages of drug candidates, drugs, and cosmeceuticals development. While each discussed approach has its own set of advantages and disadvantages, a significant shared advantage of all SCS models is their versatility in allowing the use of different lipid compositions. These lipid compositions can be tailored to mimick specific skin disorders that are challenging to reproduce in clinical studies. SCS models are appealing, because the lipid composition can be precisely defined and modified as needed to simulate the desired conditions. This flexibility enables researchers to investigate how various chemicals impact both healthy and diseased skin through subtle changes in lipid systems.

SCS systems are well suited for testing a wide range of chemical classes that may come into contact with SC lipids and potentially affect skin barrier properties negatively. Therefore, SCS can find extensive applications in the fields of skin biophysics, dermatology, transdermal drug delivery, and risk assessment.

In conclusion, future directions of the use of skin models should encopass the following main features:

(i) an increasingly mimetic composition and structure of SC ILM essential to reproduce its permeation barrier function. To this end, not only the lipid composition should be considered but also the protein fraction (e.g., keratin).
(ii) adapting skin models to high-throughput screening. The integration of skin cell-free models into microfluidic chips that mimick the skin laminar flow can boost throughput and reduce cost, cut human intervention and errors, and set a new standard for skin permeation studies.
(iii) adding a new dimension to skin permeation evaluations by providing molecular/biophysical insights into permeation, vital to predicting potential skin toxicity.
(iv) investing in models that not only mimick healthy skin but also injured skin conditions that enable researchers to delve into the intricate dynamics of how various skin conditions influence compound permeation, offering invaluable insights into treatment efficacy and safety. Mimicking permeation behaviour in diseased skin aligns with the principles of personalised medicine, emphasising tailored treatments based on individual patient profiles and needs. This would empower researchers to deepen their understanding of how specific skin conditions impact barrier function and permeability, thereby facilitating the development of precise therapeutic interventions.

## Figures and Tables

**Table 1:** The most recent examples of *Stratum Corneum* (SC) lipid model mixtures reported in the literature, from 2010 onwards, for deciphering the SC structure or as in vitro platforms to study compound–SC lipid matrix interactions.

Lipid Model Composition	Main Objective	Characterisation Techniques	Temperature (T) and pH Conditions	Main Outcomes	Ref.
Cer[AP]:Chol:PA:ChS (55:25:15:5 wt%)	Study the OA impact on bilayer architecture	Neutron diffraction	T: 32 °C pH: 9.5	OA had no effect on SC bilayer thickness, but it caused lipid disorder by interfering with alkyl chain alignment.	[104]
	Determine the electron density profile of SPP at full hydration and at different pH	Neutron diffraction	T: 25–60 °C pH: 5.0–9.0	Repeated distances of ≈41 and ≈47 Å remain at full hydration and at different pH values.	[105]
				Almost no water detected in the inter-bilayer spacing either in full or partial hydration SC states.	
	Elucidate the IPM mechanism of action as a SC permeation enhancer	Neutron diffraction	T: 32 °C pH: ND	IPM permeation results from bilayer perturbation and disordering effects. IPM induces phase separation and disturbs the multilamellar lipid assembly.	[106]
Cer[AP]:Chol:SA:ChS (55:25:15:5 wt%)	Synthesis and application of a deuterated Cer[AP]-SA in a SC lipid model	Neutron diffraction	T: 32 °C pH: ND	Deuterium label located in high-ordered lamellar structures, with a repeated distance of ≈44 Å, supporting the Cer hairpin conformation within bilayer structures previously proposed.	[107]
Cer[AP]:Chol:FFA:ChS (55:20:15:10 or 66:10:18:6 wt%) FFA = PA:SA:AA:BA:LA:CA (1.3:3.3:6.8:42.0:36.2:6.7 molar ratio)	Investigate the effects of different Chol–ChS ratios on the hydration and structure of a SC lipid model with a realistic FFA mixture	Neutron diffraction	T: 20 and 32 °C pH: ND	SC systems structure with FFA mixture is close to that of PA containing systems, with characteristic small inter-bilayer space. Ratio variation does not lead to any significant changes of membrane structure parameters. Replacement of Chol by ChS increases membrane swelling, inhibits phase separation, and promotes a more homogeneous lipid system.	[108]
Cer[AP]:Chol:SA (1:0.7:1 molar ratio)	Explore the influence of hydrophilic permeation enhancer DMSO in SC model	Neutron diffraction, PCS, IR, and leakage studies	T: 32 °C pH: 7 and 10	No specific interaction of 10% and 30% DMSO with SC model system. It was hypothesised that the permeation enhancing effect of DMSO is foreign to the SC lipid matrix.	[109]
Cer[AP]:Chol:SA (1:0.7:1 molar ratio)	Develop and optimise an oligolamellar SC model	X-ray reflectivity and FTIR	ND	New oligo-SC model has a similar total number of lipid membranes with no phase separation as native SC between corneocytes. The preparation method is reproducible.	[110]
	Study the influence of hydrophilic (urea and taurine) permeation enhancers on SC model	Neutron reflectivity, IR, SWAXS, carboxyfluorescein leakage, PCS, Langmuir isotherms, BAM, and IRRAS	T: 21 °C pH: 5.5 and 7.4	No specific interaction when 10% of urea or 5% taurine are added to SC monolayers. Permeation enhancing effect of both substances might not be related with the SC lipid matrix.	[111]
	Elucidate the IPM mechanism of action as SC permeation enhancer	Langmuir isotherms, IRRAS, BAM, and GIXD	T: 21 °C pH: 5.5	IPM showed a limited fluidising effect on the alkyl chains of SC lipids through a slight decrease in packing density of the system, irrespective of chain deuteration.	[112]
Cer[AP]:Chol:FFA:ChS (55:20:15:10 or 66:10:18:15 wt%) FFA 1 = PA:SA:AA:BA:LA:CA (1.3:3.3:6.7:41.7:36:6.7 molar ratio) FFA 2 = BA:LA:CA (8.8:7.7:1.4 molar ratio)	Study the influence of hydration and composition in the temperature behaviour of SC model	Neutron diffraction	T: 20–72 °C pH: ND	SC model with 20% of Chol and 60% humidity undergoes a phase transition in the 63–67 °C range, which, upon Chol decreasing content, shifts to larger temperatures. The hydration process increases repeated distance in 1 Å at 25 °C and in 1.6 Å at 57 °C, Phase separation occurs upon hydration at 57 °C, and the FFA-rich phase changed the repeated distance.	[113]
Cer[AS]:Chol:PA (Variable ratio)	Characterise the fluidity and microstructure of a SC lipid model	Fluorescence anisotropy and SAXS	T: 25 to 80 °C pH: 5	SC bilayers formed three kinds of lamellar structures and two kinds of hydrocarbon chain packing. Hexagonal/orthorhombic packing ratio was close to the value found in human isolated SC.	[114]
Cer[AS]:Chol:PA (26.5:13.9:59.6 mol%)	Investigate the effects of l- and d-menthols as permeation enhancers	Fluorescence anisotropy, DSC, and WAXS	T: 25 to 80 °C pH: 5	Permeation enhancing effects of optically active menthols, with d-menthol provoking stronger effects than l-menthol.	[115]
Cer[NP]:Chol:LA (1:1:1 molar ratio)	Investigate the SC model in a temperature range	Neutron diffraction and ^2^H solid-state NMR spectroscopy	T: 25–80 °C pH: ND	Coexistence of two lipid phases at 32 °C, with repeated distances of 54.2 Å and 43.0 Å. Model assembly of SC lamellar phases is proposed: the thicker phase consists of Cer[NP] in a hairpin conformation mixed with Chol and LA, while the thinner phase contains Cer[NP] in hairpin conformation.	[116]
	Explore the effect of ethanol on SC model	DSC, FTIR, and ^2^H-NMR	T: 25–75 °C pH: 5.4	Ethanol had two effects: (i) a concentration-dependent effect on the reduction of the SC phase transition temperature, and (ii) at high concentrations, it selectively extracted the FFA component from the SC lipid assemby.	[117]
Cer[NS]:Chol:BA (1:0:0 or 1:0.3:1 molar ratio)	Study the interactions between NIPAM nanogels cross-linked with MBA and SC lipid models	Neutron reflectivity and TEM	T: 24–42 °C pH: ND	Nanogels interact with the SC lipid model mainly through hydrophobic interactions, and these interactions increase with the MBA content.	[118]
Cer[NS]:Chol:(BA:LA) (1:1:1 molar ratio)	Explore the effect of various surfactants on the structure of SC model	Neutron scattering and MD	T: 32 °C pH: ND	A new mechanism for surfactant as a permeation enhancer is proposed: surfactant increases the barrier function by incorporating phase-separated Chol molecules into the SPP structure of SC membrane, while the hydrophilic surfactant headgroup remarkably increases hydration and fluidity, which eventually results in better permeability.	[119]
Cer[NS]:Chol:FFA (1:1:1 molar ratio) FFA = LA, PA or PA:SA:AA:BA:LA (1.8:4:7.6:47.8:38.9 mol%)	Examine the effect of chain length of FFAs on thermotropic phase behaviour and mixing properties of SC mixtures	FTIR and Raman imaging spectroscopy	T: 20–90 °C pH: 5	Thermotropic phase behaviour and mixing properties of lipid mixtures strongly depend on chain length and its hydration state. While a complex mixture of FFA and single LA did not significantly change the SC mixture phase behaviour, in mixtures containing only PA, phase separation was observed after hydration and long-term storage. Evidence that matching the FFA chain length and Cer acyl chain promotes mixing.	[120]
Cer[NdS]:Chol:PA (1:1:1 molar ratio) D-*erythro*- or L-*threo*-[NdS]	Investigate the contribution of stereochemistry of Cer[NdS] in SC model	DSC and SWAXS	T: 20–180 °C pH: ND	Inter- and intramolecular interaction was greater in the D-*erythro*-based system than in the L-*threo*-based ones, resulting in appreciable differences in lamellar periodicities. Stereostructure of Cer molecules can be determinant in lamellar periodicities.	[121]
Cer[AP]/Cer[NS]:Chol:SA (1:1:1 molar ratio)	Develop an IR spectroscopy approach for studying lateral phase separation and lamellar structure formation in SC models	FTIR	T: 31 °C pH: 5.5	It was possible to detect the reorganisation of Cer amide H-bonds from disordered to ordered forms, appearance of ordered Cer chain orthorhombically packed, and the phase separation of orthorhombically packed domains of SA with the H-bonded dimeric headgroup. Kinetic IR spectroscopy measurements showed to be promising in monitoring the temporal sequence for phase separation and lamellar structure formation.	[122]
Cer:Chol:PA (1:1:1 molar ratio) Cer = [AS], [NP], [NP]-SA or [NS]	Investigate the structure of hydrated SC lipid model	Mid-IR spectroscopy	T: 30–35 °C pH: 5.5	Between 30 and 35 °C, orthorhombic and hexagonal chain packing are observed, which favoured the formation of orthorhombic packing with decreased hydration. Hydrated Cers exhibited high solid crystalline- to liquid crystalline-phase transition temperatures. At low temperature, phase separation is observed: a Cer-PA domain and a PA-Chol domain. Cer[NS]-SA improved the system miscibility. Formation of distinct crystalline-like domains embedded in a more fluid phase supported the domain mosaic model. Headgroup architecture of Cer mainly determines the structure of hydrocarbon chains, favouring the orthorhombic perpendicular subcell lattice.	[123]
Cer[AP]/Cer[NP]:Chol:SA (55:25:20 wt%)	Investigate the lamellar structure of SC model	Neutron diffraction and NMR	T: 32 °C pH: 7.4	Absence of only one OH group caused drastic structural changes, supporting the great influence of the Cer headgroup in the structure of the SC membrane. Cer[NP]-based system exhibited higher lamellar order than the Cer[AP] system, with completely different temperature-dependent behaviour and hydration characteristics. Cer[NP] was able to prevent the model membrane from significant hydration and swelling, due to intense intra- and intermolecular headgroup interactions.	[42]
Cer[EOS]:Chol:FFA (Variable ratio) FFA = PA:SA:AA:BA:TA:LA:CA (1.8:4:7.7:42.6:5.2:34.7: 4.1 molar ratio)	Investigate the molecular assembly and lipid packing in SC model	SAXS	T: 20–100 °C pH: ND	Equimolar ratio forms only one lamellar phase with a long repeated distance of 14.7 nm, with FFA and Cer chains undergoing phase transition in different temperature ranges, while part of their chains are mixing in orthorhombic lattice. Although deviations in the FFA content are less critical for formation of the 14.7 nm lamellar phase than the Chol content, a certain degree of FFA chain length is important.	[124]
Cer[AdS]:Chol:PA (Variable ratio)	Examine the quantitative effect of Chol reduction in SC model	Raman, DSC, PXRD, and SAXS	T: 20–120 °C pH: ND	Amount of Chol in the SC model was inversely correlated with the hexagonal structure ratio (RHex/Ort) in the packing structure. RHex/Ort values are highly correlated with the amount of lipid elution effect of surfactants.	[125]
Cer[NS16]/Cer[NS24]:Chol:FFA:ChS (1:1:1 molar ratio + 5 wt%) FFA = LA or PA:SA:AA:BA:LA (1.8:4:7.6:47.8:38.8 wt%)	Investigate the membrane behaviour of long and very long Cer[NS] in SC model	FTIR	T: 28–100 °C pH: 5.5	While very long Cer[NS24] preferred an extended conformation in which FFAs (either LA or heterogeneous mixture) were associated with Cer chains, shorter Cer[NS16] were less FFA miscible, being mostly phase separated.	[126]
Cer[NS16]/Cer[NS24]:Chol:FFA (1:1:1 molar ratio) FFA = PA, LA, AA or LA:AA:SA:PA (84.6:8.75:4.57:2.05 mol%)	Study the influence of acyl chain length on lipid mixing properties in SC model	Raman and IR	T: 33 °C pH: 5	The combination of Cer and FFA bearing very long chains is required for the formation of homogeneous mixtures, while shorter chained FFAs lead to micro-sized domains.	[127]
_BB_Cer[NP]:Chol:SA (1:1:1 or 0.5:1:1 molar ratio)	Investigate the thermotropic and kinetics of lipid dynamics and domain formation in normal and Cer deficient SC model	FTIR	T: 19–85 °C pH: 5.5	Kinetic and thermotropic studies displayed differences in both Cer and FFA chain fluidity and reduction in Cer levels had an ordering effect. In a Cer-deficient model, the formation of Cer orthorhombic domains is retarded, affecting the lipid barrier structure.	[128]
_BB_Cer:Chol:SA (1:1:1 molar ratio)	Study the water vapor uptake and surfactant sorption onto SC model	QCM	T: 22 °C pH: ND	Surfactant sorption was found to be concentration-dependent even beyond its critical micelle concentration. QCM results are in accordance to reported clinical data, which suggest that this approach is promising to monitor the barrier properties of the SC model.	[129]
DPPC:Chol (7:3 molar ratio) Cer[AP]:Chol:SA (14:10:14 molar ratio)	Explore the effect of a natural and four synthetic surfactants on two SC monolayer models	Langmuir isotherms, surface dilatational rheology, and fluorescence microscopy	T: 21 °C pH: ND	The four synthetic surfactants were capable of solubilising lipids from the SC monolayer. Natural saponin-rich extracts increased surface pressure greater than synthetic ones and enhanced SC elastic properties. No evidence of SC lipids removal by saponin-rich extract suggests a surfactant action through changes in the physical state of the monolayer.	[130]
Cer[EOS]:Cer[AP]: Chol:BA (23:10:33:33 wt%)	Study the effect of IPM in the SC lipid model assembly	Neutron diffraction	T: 32, 50 and 70 °C pH: ND	IPM had a disordering effect in the rigid lamellar structure and influenced the phase behaviour of SC model possibly by interaction with lipid headgroups. IPM prevented the formation of LPP by favouring the SPP formation and decreasing SC barrier properties.	[131]
Cer[EOS]:Cer[AP]: Chol:PA:ChS (30:30:20:15:5 wt%)	Characterise the LPP SC lipid model in excess water	SAXS	T: ND pH: 7.2 or 9	In excess water at pH 7.2, SC lipid membrane revealed two SPP with repeated distances of 47 and 35.7 Å and a LPP with a repeated distance of 127 Å. Increasing the pH to 9 resulted in the destruction of LPP and in a single SPP with repeated distance of 48.3 Å.	[132]
Cer:Chol:SA+ChS (1:1:1 molar ratio + 5 wt%) Cer = [NP]:[AP], [NP]:[NS], [AP]:[NS] or [NP]:[AP]:[NS] (1:1 or 1:1:1 molar ratio)	Characterise each Cer role in the SC membrane: effects on assembly, miscibility, and thermotropic behaviour	SWAXS and IR spectroscopy	T: 20–80 °C pH: ND	Cer[NP] showed the ability to establish very strong H-bonds within its solid crystalline structure, which results in a phase separation effect and orthorhombic lattice that weakens when Cer[NP] is combined with the other two Cer types. The importance of acyl chain length was demonstrated, and it was clear that even a small variation in the Cer headgroup structure or minor change in the membrane composition can lead to great differences in the phase behaviour of the SC model system.	[133]
Cer[NS]:Cer[EOS]:FFA:Chol:ChS (Variable ratio) FFA = PA:SA:AA:BA:LA (1.8:4:7.6:47.8:38.8 mol%)	Investigate the lamellar structure in SC model	SAXS	ND	In the SC model, medium and very long lamellar phases may be formed in the absence of Cer[EOS], whereas these Cer likely stabilise the final lipid assembly of LPP by riveting the adjacent lipid layers. An increased ChS:Chol molar ratio modulated the membrane polymorphism.	[134]
Cer[NP]:Cer[AP]:Chol:FFA (Variable ratio)	Understand the SC lipid matrix assembly	Neutron Diffraction and MD	ND	Except for Chol, all lipids showed a strong H-bonding network, which is correlated with tighter lipid packing. A strong H-bonding network is determinant in SC packing, but the location of -OH groups is equally important.	[135]
Cer[EOS]:Cer[NS]: Chol:FFA (variable ratio) FFA = PA:SA:AA:BA:LA (4:8:48:40 mol%)	Investigate the effects of replacing Cer[NS] by Cer[EOS]	SAXS and solid-state and diffusion NMR	T: 32 °C pH: ND	SC model is assembled in the LPP phase, where the rigid headgroup of Cer[EOS] is anchored at the lamellar interfatial region, and the isotropic fluid middle and terminal segments of linoleate chains of Cer[EOS] show slow translational diffusion. Unusual self-assembled structure demonstrated a strong impact on barrier function.	[136]
Cer[NP]:*D*-/*L*-Cer[AP]:Chol:LA (0.66:0.34:0.7:1 or 0:1:0.7:1 molar ratio)	Study the influence of the Cer[AP] conformation on the lamellar structure of SC model	Neutron diffraction and SAXS	T: 32 °C pH: ND	Strong influence of Cer[AP] conformation on lamellar and nanostructure of the multilamellar SC system. While D- alone showed a crystalline-like behaviour, L- presented no lamellar phase. When combined with Cer[NP], the D- based system adopted a more native-like structure, while L- promoted a very crystalline-like behaviour.	[137]
Cer[EOS]:Cer[NS]: Chol:PA (Variable ratio)	Investigate the effects of vaporised *L*-menthol on SC lipid matrix	DSC, SAXS, and ATR-FTIR	T: 20–120 °C pH 5	SC mixture formed characteristic orthorhombic and hexagonal packing structures. *L*-menthol showed the ability to act as a permeation enhancer through a liquid crystalline lamellar structure by affecting the Cer carbon chain and dissociating the H-bond hydrophilic groups.	[138]
Cer[EOS]:Cer[NS]: Chol:FFA (0.4:0.6:1:1 molar ratio) FFA = LA:BA:SA:PA (84.6:8.75:4.57:2.05 molar ratio)	Characterise the chain order of Cer[EOS] in SC model forming LPP	SAXS, H-NMR, and IR	T: 25–70 °C pH: 5	LPP structure is formed by a solid ordered phase in which highly disordered liquid domains formed by the oleate chain of Cer[EOS] are embedded. Cer[EOS] acts as a strong modulator of SC fluidity balance.	[139]
Cer[NS]:Cer[AP]:Chol:LA (variable ratio)	Explore the impact of Cer[NS] on SC model structure	Neutron diffraction and MD	T: 32 °C pH: ND	Both Cers showed a similar influence on lamellar structure and promoted lipid miscibility with Chol; The SC model composed of [NS]/[NP] 2:1 and 1:2 presented repeated distances similar to those of natural-like SPP.	[140]
Cer[NP]:Cer[AP]:Chol:ChS:LA:PA:SA (33:22:25:5:7.5:3.75:3.75 mol%)	Investigate the effect of model CPEs on SC liposomes	Sodium fluorescein leakage and fluorescence anisotropy	T: 25 °C pH: 9	SC liposomes as promising models for high-throughput screening of permeation enhancers effectivity.	[141]
	Investigate the potential of SC liposomes for CPEs permeation study (ethanol as model) and comparison with animal skin permeation studies	Fluorescein leakage, fluorescence anisotropy, animal skin impedance, and hairless rat skin permeation	T: 25 °C pH: 9	Good correlation between permeation in SC liposomes and in hairless rat skin. Ethanol action on SC lipids and permeation-enhancing effect mainly occurs through polar headgroup perturbation.	[37]
Cer:Chol:LA (1:1:1 molar ratio) Cer = [NS]:[NH] or [NS]:[AP] (1:1 molar ratio)	Understand the basic structure of SC membranes	NMR, DSC, and WAXS	T: 20–120 °C pH: 5.5	Coexistence of several phases at all temperatures tested: at skin temperature, molecules formed a solid crystalline phase with orthorhombic lipid packing and only a small fraction of lipids is in a liquid crystalline state. While the Cer[AP]-based SC system formed a lamellar phase with SPP, the Cer[NH]-containing system formed a lamellar phase with 10.7 nm periodicity.	[142]
Cer[AP]:_br_Cer[EOS]: Chol:BA (10:23:33:33 wt%)	Explore the localisation of _br_Cer[EOS] species within LPP of SC model	Neutron diffraction	ND	SC lipid mixture formed a LPP-sized by 114 or 118 Å with two coexisting SPP of 48 Å and 45 Å. Cer[EOS] was found in LPP and absent in SPP, while CER[AP] was found in both short phases but not within the LPP.	[143]
_br_Cer[EOS]:Cer[AP]: Chol:BA (23:10:33:33 wt%)	Synthesis of new artificial Cer[EOS] species and implication to SC model structure	DSC, FTIR, Raman, Neutron diffraction, and MD	T: 20–120 °C pH: ND	Synthesised _br_Cer[EOS] showed to be promising in substituting native form in terms of the formation of well-ordered lipid bilayers with a lipid assembly equivalent to the model matrix comprising native Cer[EOS].	[144]
Cer[NS]:Cer[AS]:Chol:PA:ChS (25:15:25:25:10 wt%)	Understand the nature of Posintro^TM^ permeation into SC lipid matrix	FRET, ITC, AFM, Cryo-TEM, and EIS	T: 5, 15, 25 or 37 °C pH: 7.4	Posintro^TM^ nanoparticles disturbed the SC bilayers in a DC-Chol content-dependent manner. Nanoparticle permeation into skin may be involving their fusion with the SC lipid matrix.	[145]
Cer[EOS]:Cer[NS]: Chol:FFA (0.4:0.6:1:1 molar ratio) FFA = PA:SA:AA:BA:LA (1.8:4:7.6:47.8:38.8 mol%)	Study the interaction between Cer[NS] and FFA chains in LPP	FTIR and SAXS	T: 0–90 °C pH: ND	Cer[NS] acyl chain and FFAs are mixed and colocalised within central region of LPP. Cer[NS] adopted an extended conformation that increases the packing density, inhibits membrane swelling, and allows flexibility of the SC system.	[146]
Cer[NP]:Cer[AP]:Chol:LA (0.66:0.34:0.7:1 or 0.34:0.66:0.7:1 molar ratio)	Investigate the influence of Cer[NP] and Cer[AP] on SC nanostructure	Neutron diffraction	T: 32–37 °C pH: ND	SC mixture showed similar thickness to the SPP native SC lipid matrix, with a repeated distance of 5.45 nm. Cer[AP] are assembled in such a way that its C24 chain is somehow shorter than the C24 chain of Cer[NP], not overlapping as much with the opposite lamellar leaflet. In the [NP]:[AP] 2:1 ratio system, chains are mostly straight-packed, and C24 chains show a broad overlapping region in the lamellar midplane.	[147]
Cer[NS]:Cer[EOS]: Chol:FFA (0.6:0.4:1:1 molar ratio) FFA = LA:AA:SA:PA (84.6:8.75:4.57:2.05 mol%)	Study the lipid spatial distribution of SC model	Raman spectroscopy and AFM-IR	T: 33 °C pH: 5.0	SC model showed to be overall homogeneous with small, slightly enriched, and depleted regions in a lipid component. Fluid nature of the Cer[EOS] chain was confirmed, while the rest of the lipid matrix was highly ordered, supporting the three-layer model for the SC structure.	[148]
Cer[EOS]:Cer[NS]:SP: SA:Chol:LA (Variable ratio)	Determine the effect of Cer reduced concentration on LPP structure	Neutron diffraction, SAXS, and FTIR	T: 23 or 37 °C pH: 5.0	LPP structure remained undisturbed at least until the replacement of 25% of Cer[NS]. An increased ceramidase activity or reduced Cer synthase activity does not lead to dramatic changes in the lipid phase behaviour of Cers. With increased FFA concentration, Cers in LPP tend to redistribute.	[149]
Cer[NP]:Cer[AP]:_br_Cer[EOS]:Chol:LA (0.6:0.3:0.1:0.7:1 molar ratio)	Examine the lamellar and nanostructure of SC model	Neutron diffraction	T: 32 °C pH: ND	The SC mixture was able to mimick natural SPP with a lamellar repeated distance of 5.47 nm, in which LPP formation was not detected. Cer[EOS] was integrated into SPP spanning multiple layers.	[150]
Cer[EOS]:Cer[NS]: Cer[NP]:Chol:LA (0.4:0.3:0.3:1:1 molar ratio)	Examine the location of Cer[NP] and Cer[NS] in LPP unit cell	Neutron diffraction	T: 37 °C pH: 5.0	In LPP of a SC model system presenting a repeated distance of 12.6 nm, the acyl chain of Cer[NP] was predominantly located in the central part of the trilayer structure, with a minor fraction at the unit cell boundary, similar to Cer[NS]. Both Cers adopted a linear conformation in the LPP unit.	[151]
Cer:Chol:FFA (1:1:1 molar ratio) Cer = [NS]:[NP24]:[AS]: [NP16]:[AP] (60:19:5:11:6 molar ratio) FFA = PA:SA:AA:BA:TA:LA:CA (1.8:4:7.7:42.6:5.2:34.7: 4.1 molar ratio)	Study the molecular assembly of SPP	Neutron diffraction	T: 25 °C pH: 5.0	SPP were characterised with a repeated distance of 5.4 nm in a typical bilayer structure. Cers, with interdigitating acyl chains, were symmetrically assembled in the centre of the unit cell.	[152]
	Determine the FFA and Chol location into SPP of SC model	Neutron diffraction	T: 25 °C pH: ND	SC model was assembled in the lamellar phase with a repeated distance of 53.9 Å. Chol headgroup was found to be somewhat inward from the unit cell boundary, with its tail located 6.2 Å from the unit cell centre. FFA headgroups were located at the unit cell boundary, with their acyl chains encircling the core of the unit cell.	[153]
Cer:Chol:FFA (1:1:1 molar ratio) Cer = [EOS]:[NS]:[NP]:[AS]: [NP16]:[AP] or [EOS]:[NS] (39:38:10:3:6:4 or 40:60 mol%) FFA = PA:SA:AA:BA:TA:LA:CA (1.8:4:7.6:47.8:38.8 mol%)	Explore both assembly and conformation of Cer[NS] within LPP unit cell	Neutron diffraction and SAXS	T: 25 and 32 °C pH: ND	In the repeating trilayer LPP unit, the acyl chain of Cer[NS] was located in the central and outer layers, while sphingosine chain was located exclusively in the middle of the outer layers. Cer[NS] showed the same behaviour regardless the Cers mixture studied.	[154]
Cer:Chol:FFA (1:0–1:0/1 molar ratio) Cer = [EOS]:[NS]:[NP24]: [AS]:[NP16]:[AP] (0:60:18:5:10:7 or 40:36:11:3:6:4 mol%) FFA = PA:SA:AA:BA:TA:LA:CA (1.8:4:7.7:42.6:5.2:34.7: 4.1 mol%)	Explore the effect of Chol on lipid assembly in each lamellar phase	FTIR and SAXS	T: 20–60 °C pH: 5.0	A minimum of 0.2 Chol in 1:0.2:1 molar ratio mixture was required for the formation of each lamellar phase, and its gradual increment increased the fraction of lipids forming very dense orthorhombic lateral packing. Chol revealed to be an important building block for the formation of orthorhombic lateral packing of lipid chains and to induce the formation of both SPP and LPP.	[155]
Cer:Chol:FFA (1:1:1 molar ratio) Cer = [EOS]:[NS]:[NP24]: [AS]:[NP16]:[AP] (40:60:0:0:0:0 or 40:36:11:3:6:4 mol%) FFA = PA:SA:AA:BA:TA:LA:CA:MA (1.8:4:7.7:42.6:5.2:34.7: 4.19 mol% with increased amount of PA)	Investigate the effects of FFA chain lengths (C16–C28) on lamellar phase and lateral packing of SC model	SAXS, FTIR, and transepidermal water loss measurements	T: 0–90 °C pH: 5.0	Alterations in LPP behaviour are not strictly correlated with chain length, and shorter FFA chain length models exhibit a different behaviour compared to their longer counterparts. Only models FFA C20 and longer have orthorhombic phase transition temperatures that increase with the increasing chain length. LPP is a robust structure that can withstand a certain threshold of change before its barrier function is affected.	[156]
Cer:Chol:FFA (1:1:1 molar ratio) Cer = *L*-/*O*-/*S*-[EOS]: [NP]:[NS24]:[AS]: [NS16]:[AP] (30:42:13:3.4:7.5:4.1 mol%) FFA = PA:SA:AA:BA:TA:LA:CA (1.3:3.2:6.9:42:5.3:37:4.7 mol%)	Study the influence of Cer[EOS] moiety on SC lipid assembly	FTIR and SAXS	T: −9.5–90 °C pH: 5.0	While Cer *S* stabilised the orthorhombic phase, *L*- and *O*-forms promoted the presence of LPP. The copresence of the three Cer[EOS] may contribute to a proper skin barrier function.	[157]
Cer:Chol:FFA (1:1:1 molar ratio) Cer = [EOS]:[NS]:[NP24]: [AS]:[NP16]: [AP] (13.3:12:3.7:1:1:1.3 mol%) FFA = PA:SA:AA:BA:TA:LA:CA (0.6:1.3:2.6:14.2:1.7:11.5:1.4 mol%)	Study the molecular assembly of LPP in the SC	Neutron diffraction	T: 25 °C pH: 5.0	SC lipid mixtures with high content of Cer[EOS] formed LPP with a repeated distance of ≈130 Å in a trilayer structure with Cer[NS] and LA interdigitated mostly at the unit cell.	[158]
Cer:Chol:FFA (1:1:1 molar ratio) Cer = [EOS]:[NS]:[NP24]: [AS]:[NP16]:[AP] (13.3:12:3.7:1:2:1.3 mol%) FFA = PA:SA:AA:BA:TA:LA:CA (0.6:1.3:2.6:14.2:1.7:11.5:1.4 mol%)	Determine the lipid components’ location in the LPP unit cell	Neutron diffraction	T: 25 °C pH: ND	A molecular model is proposed for the LPP unit cell with repeated distance of ≈129 Å: (i) Chol is located in two outer layers of LPP (at ≈26 Å from the cell unit centre), (ii) Cer[EOS] (at ≈21.4 Å from the cell unit centre) is linking two outer layers with the central lipid layers, and (iii) the two other lipid subclasses are predominantly located in the central layer of LPP.	[159]
Cer[NS18]/Cer[NP18]: SA:Chol (1:1:1 molar ratio)	Explore the chain-matched and headgroup influence on SC assembly	^2^H-NMR	T: 32, 50 and 75 °C pH: ND	Mixtures containing Cer[NP] tend to create more fluid phases than Cer[NS]: the extra OH of Cer[NP] generates a steric conflict when mixed with chain-matched SA and Chol, which destabilises the orthorhombic lattice of SC lipid mixtures and favours the production of liquid crystalline phases.	[160]
Cer[EOS]:Cer[NS]: Chol:FFA (0.3:0.7:0.45:1 molar ratio) FFA = PA:SA:AA:BA:LA (1.8:3.9:7.5:47.8:39 wt%)	Study the Cer[EOS] molecular behaviour in a SC lipid model	SAXS, WAXS, ^2^H-NMR, and Neutron diffraction	T: 32 °C pH: 5.4	LPP structure with alternating fluid (sphingosine chain-rich), rigid (acyl chain-rich), isotropic (linoleate-rich), rigid (acyl-chain rich), and fluid layers (sphingosine chain-rich).	[161]

AA—arachidonic acid; AFM—atomic force microscopy; ATR-FTIR—attenuated total reflection Fourier-transform infrared spectroscopy; BA—behenic acid; BAM—Brewster angle microscopy; _BB_Cer—bovine brain ceramide; _br_Cer—branched ceramide; CA—cerotic acid; Cer—ceramide; Cer[AdS]—α-hydroxy fatty acid/dihydrosphingosine base ceramide; Cer[AP]—α-hydroxy fatty acid/phytosphingosine base ceramide; Cer[EOS]—ester-linked ω-hydroxy fatty acid/sphingosine base ceramide; Cer[NdS]—non-hydroxy fatty acid/dihydrosphingosine base ceramide; Cer[NH]—non-hydroxy fatty acid/6-hydroxy-sphingosine base ceramide; Cer[NP]—non-hydroxy fatty acid/phytosphingosine base ceramide; Cer[NS]—non-hydroxy fatty acid/sphingosine base ceramide; Chol—cholesterol; ChS—cholesteryl sulfate; CPE—chemical permeation enhancer; DMSO—dimethyl sulfoxide; DPPC—dipalmitoylphosphatidylcholine; DSC—differential scanning calorimetry; EIS—electrochemical impedance spectroscopy; FFA—free fatty acid; FRET—Förster resonance energy transfer; FTIR—Fourier-transform infrared spectroscopy; GIXD—Grazing incidence X-ray diffraction; IPM—isopropyl myristate; IR—infrared; IRRAS—infrared reflection absorption spectroscopy; ITC—isothermal titration calorimetry; LA—lignoceric acid; LPP—long periodicity phase; MA—montanic acid; MBA—N’-methylenebisacrylamide; MD—molecular dynamics; ND—not discriminated; NIPAM—n-isopropylacrylamide; NMR—nuclear magnetic resonance; OA—oleic acid; PA—palmitic acid; PCS—photon correlation spectroscopy; QCM—quartz–crystal microbalance; SA—stearic acid; SAXS—small-angle X-ray scattering, SWAXS—small and wide angle X-ray scattering; SC—*Stratum Corneum*; SP—Sphingosine; SPP—short periodicity phase; TA—tricosylic acid; TEM—transmission electron microscopy; UV—ultraviolet; WAXS—wide-angle X-ray scattering; wt—weight.

**Table 2:** Published approaches of *Stratum Corneum* surrogates (SCS) incorporating SC lipid biomimetic models for permeation studies.

SC Lipid Model Composition	Porous Substrate	Characterisation Techniques	Model Compounds	Main Outcomes	Ref.
**Airbrush approaches**					
Cer:Chol:FFA (1:1:1 mol ratio) Cer = [EOS]:[NS]:[NP24]: [AS]:[NP16]:[AP] (15:51:16:4:9:5 molar ratio) FFA = PA:SA:AA:BA:TA:LA:CA (1.3:3.3:6.7:41.7:5.4:36.8:4.7 molar ratio)	Polycarbonate 50 nm filter	SWAXS and CryoEM	n.a.	Airbrush is able to homogeneously spray a SC mixture onto a polycarbonate filter. Synthetic lipids in the SCS resembled the orientation of the ILM of SC: the width of the arcs of SCS were close to the ones in the SC. SCS characteristics showed great potential for use as a skin barrier diffusion model.	[58]
		Diffusion studies	PABA, ethyl-PABA, and butyl-PABA	Barrier properties of SCS prepared with a 12-μm-thick lipid layer are similar to those of isolated human SC. SCS model can be easily tuned by adjusting the lipid mixture to mimick different healthy and diseased skin.	[30]
Cer:Chol:FFA (1:1:1 molar ratio) Cer = [EOS]:[NS]:[NP24]: [AS]:[NP16]:[AP] (15:51:16:4:9:5 molar ratio) FFA = PA:SA:AA:BA:TA:LA:CA (1.8:4:7.7:42.6:5.2:34.7:4.1 molar ratio)	Polycarbonate 50 nm filter	Different airbrush methods, SAXS, and diffusion studies	Benzoic acid	Irrespective of the preparation method, benzoic acid permeation profiles were very similar to human SC. While the rotor method is claimed to increase efficiency and reproducibility, the Linomat method reduced the lipid loss during lipid airbrush and was thus selected as the best method to spray lipids on porous substrate.	[79]
Cer:Chol:FFA:ChSO_4_ (Variable ratio) Cer = [EOS]:[NS]:[NP24]: [AS]:[NP16]:[AP] (15:51:16:4:9:5 molar ratio) FFA = PA:SA:AA:BA:TA:LA:CA (1.8:4:7.7:42.6:5.2:34.7:4.1 mol%)	Polycarbonate 50 nm filter	FTIR, SAXS, and permeability studies	Benzoic acid	Two SCS models showed significant changes in the benzoic acid steady-state flux: an excess of Chol decreased flux, while an excess of ChSO_4_ increased the flux. Lipid composition disorders in psoriasis and dry skin may not be responsible for impaired skin barrier function in vivo.	[171]
Cer:Chol:FFA (1:1:1 molar ratio) Cer = [EOS]:[NS]:[NP]:[AP] (15:51:16:4:9:5 molar ratio) FFA = PA:SA:AA:BA:TA:LA:CA or MyA:PA:SA:AA:BA (Variable ratio)	Polycarbonate 50 nm filter	SAXS, EM, and permeation studies	Benzoic acid	Permeability of the SC system to benzoic acid closely follows that of human SC at temperatures ranging from 31 to 43 °C.	[172]
Cer[AP]:Chol:PA:ChS (55:25:15:5 wt%)	Polycarbonate 50 nm filter	SAXS, polarisation, confocal and ESEM microscopies, Raman imaging, FTIR, and diffusion studies	Urea	A continuous uniformly distributed lipid layer on polycarbonate filters was obtained. Membranes showed higher barrier function than isolated human SC.	[173]
Cer:Chol:FFA (1:1:1 molar ratio) Cer = [EOS]:[NS]:[NP24]:[NP16] (16:56:18:10 mol%)	Polycarbonate 50 nm filter	FTIR and interaction studies	γ-undecalactone, DDA, and diethyl 1,4-cyclohexanedicarboxylate	The dose-dependent effects of the chemicals on the lateral molecular packing in the SCS were qualitatively identical to those observed by IR spectroscopy in human SC.	[174]
Cer[NS]:Chol:PA (1:1:1 molar ratio)	Polycarbonate 50 nm filter	Laurdan fluorescence, SEM, Raman scattering, and permeability studies	Benzoic acid and CAF	SCS showed continuous coverage of gel-phase lipids, allowing it to be used in permeability studies. Barrier properties in the same order as ‘real’ SC.	[175]
Cer:Chol:FFA (1:1:1 molar ratio) _pig_Cer or _syn_Cer = [EOS]:[EOP]:[NS]:[NP24]: [AS]:[NP16]:[AP] (Variable ratio) FFA = PA:SA:AA:BA:TA:LA:CA (1.8:4:7.7:42.6:5.2:34.7:4.1 mol%)	Polycarbonate 50 nm filter	SAXS, FTIR, and permeation studies	HC	Reduced barrier in SCS prepared with _pig_Cers compared to synthetic Cers. Chain length distribution affects the lipid barrier.	[164]
Cer:Chol:FFA (1:1:1 molar ratio) Cer = [EOS]:[NS]:[NP24]: [AS]:[NP16]:[AP] (15:51:16:4:9:5 molar ratio) FFA = PA:SA:AA:BA:TA:LA:CA (1.8:4:7.7:42.6:5.2:34.7:4.1 mol%)	Polycarbonate 50 nm filter	SAXS, FTIR, TEWL, and permeation studies	HC	Presence of MUFAs in the FFA fraction enhanced SCS permeability by inducing the formation of hexagonal lateral packing. Lipid barrier function decreased with the increased unsaturation degree.	[176]
_a_Cer:Chol:LA:ChS (1:1:1 molar ratio + 5 wt%)	Polycarbonate 15 nm filter	IR, SWAXS, and permeation studies	TH and IND	Strong permeabilisation effects of pentadecasphingosine Cer with short acyls. SCS permeability to relatively small and hydrophilic compounds was more sensitive to lamellar assembly than to the overall lipid chain order and packing.	[177]
Cer:Chol:FFA (1:1:1 molar ratio) Cer = [NS] or [NS]:[NP24]:[AS]:[NP16]: [AP] (60:19:5:11:6 mol%) FFA = PA:SA:AA:BA:LA(1.8:4:7.6:47.8:38.9 mol%)	Polycarbonate 50 nm filter	FTIR, XRD, and permeability studies	ethyl-PABA	Ethyl-PABA flux was affected by the SCS FFA composition: flux was higher when a mixture of FFA was used than when a single FFA C24 was present. Ethyl-PABA flux was not affected by the Cer composition.	[178]
Cer:Chol:SA:ChS (1:1:1 molar ratio + 5 wt%) Cer = [NP]:[AP] (Variable ratio)	Polycarbonate 15 nm filter	IR, EI, EM, and permeation studies	TH and IND	SCS based on one Cer showed greater permeability for both chemicals (Cer[AP] more than Cer[NP]) than those based on their mixture, without significant difference from the flux on porcine skin.	[179]
Cer:Chol:FFA (1:1:1 molar ratio) Cer = [EOS]:[NS], [EOS]:[AS], [EOS]:[NP] or [EOS]:[AP] (40:60 mol%) FFA = PA:SA:AA:BA:LA (1.8:4:7.6:47.8:38.8 mol%)	Polycarbonate 50 nm filter	FTIR, X-ray, and permeability studies	ethyl-PABA	Due to distinct in-phase behaviour, lipid chain packing, and headgroup interactions, sphingosine-based Cer SCS resulted in a higher permeability than the phytosphingosine SCS.	[169]
Cer:Chol:FFA (1:1:1 molar ratio) Cer = [EOS]:[NS]:[NS24]: [NS16]:[NP]:[AS24]: [AS16]:[AP24] (Variable ratio) FFA = PA:SA:AA:BA:LA (Variable ratio)	Polycarbonate 50 nm filter	FTIR, SWAXS, permeability studies, and TEWL	ethyl-PABA	Changes in the SCS lipid compositions mimicking atopic dermatitis patients resulted in decreased barrier capability; FFA compositional changes showed a greater impact on barrier function than changes in the Cer profile.	[180]
_h_Cer:Chol:FFA:ChS (1:1:1 molar ratio + 5 wt%) FFA = PA:SA:AA:BA:LA (1.8:4:7.6:47.8:38.8 mol%) Cer[NS]:Cer[EOS]:Chol: LA:ChS (Variable ratio)	Polycarbonate 15 nm filter	WAXS, FTIR, TEWL, electrical impedance, and permeation studies	TH and IND	While 40% of the Chol SCS was less permeable than the control, Chol depletion did not compromise the barrier function to water, TH, and IND.	[181]
_a_Cer[NS]:Chol:LA:ChS (1:1:1 molar ratio + 5 wt%)	Polycarbonate 15 nm filter	XRD, IR spectroscopy, permeability studies, water loss, and electrical impedance	TH and IND	SCS containing 3-deoxy-Cer, as well as 1-deoxy-Cer, had similar permeabilities. Methylation of the Cer[NS] nitrogen improved its miscibility with Chol, disordered lipid chains, and increased the permeability compared to the physiological Cer[NS] control.	[36]
Cer:Chol:LA (1:1:1 molar ratio) Cer = [EOS]:[NS24] or [EOS:NS16] (Variable ratio)	Polycarbonate 50 nm filter	FTIR, SAXS, and permeation studies	ethyl-PABA	Ethyl-PABA permeability increased significantly when ≥50% of Cer[NS24] was substituted with Cer[NS16].	[182]
Cer:Chol:FFA+ChS (1:1:1 molar ratio + 5 wt%) Cer = [NS], *R*-/*S*-[AS], [NdS], *R*-/*S*-[AdS], [NP] or *R*-/*S*-[AP]	Polycarbonate 15 nm filters	XRD, FTIR, and permeability studies	TH and IND	Mixed effects highly dependent on the sphingoid base chain, configuration, and permeability marker used.	[37]
Cer:Chol:FFA (1:1:1 molar ratio) Cer = [EOS]:[NS] (10–90:90–10 mol%) FFA = PA:SA:AA:BA:LA (1.8:4:7.6:47.8:38.8 mol%)	Polycarbonate 50 nm filters	SAXS, FTIR, permeation studies, and TEWL	ethyl-PABA	Steady-state flux only increased when the Cer[EOS] content was raised to 50%, becoming significantly higher when it was raised to ≥70%.	[183]
Cer:Chol:FFA:ChS (1:1:1 molar ratio + 5 wt%) Cer = [EOP]:[EOdS]:[EOS]: [AP]:[AS]:[NP]:[NS]: [NdS]:[AdS] (Variable ratio) FFA = BA:LA:AA:SA:PA (47.1:41.4:6.9:3.3:1.3 mol%)	Polycarbonate 15 nm filter	XRD, FTIR, electrical impedance, and permeability studies	TH and IND	SCS with 10–20 mol% of acylCers mimick the nanostructure and permeability of healthy skin barrier lipids, while 0–5 mol% mimick some hallmarks of a diseased skin lipid barrier.	[184]
				Permeability studies indicated that ω-OHCers could not be substituted by ω-O-acylCers. Absence of ω-O-acylCers resulted in diminished barrier properties.	[185]
Cer[NS]:Chol:FFA:ChS (Variable ratio) FFA = PA:SA:AA:BA:LA (1.8:3.9:7.5:47.8:39 mol%)	Polycarbonate 15 nm filters	H-NMR, SAXS, WAXS, zeta potential, and permeability studies	TH and IND	High ChS:Chol ratio increased the SCS permeability.	[186]
Cer[NS]:Chol:PA (1:1:1 molar ratio)	Polycarbonate 50 nm filters	SEM, DLS, and permeation studies	CAF	CAF showed similar and comparable permeability profiles in SCS using two different approaches and in pig skin.	[187]
**Skin-PAMPA^TM^ approaches**					
Certramides:Chol:SA (0 to 1:1:1 molar ratio)	Multiscreen-IP 450 nm pores	Permeability studies	CIP, NFD, and VER	A relationship between the SCS concentration of certramides (from 0 to 100%) and the permeability of the compounds was found. Strongest effect on permeability was caused by the certramide C12–C16.	[80]
Certramides:Chol:SA: Si oil	Stirwell^TM^ PAMPA	Permeability studies	APAP, DCF, FUR, NAP, PEF, TH, and VER	By comparison to three different databases, skin–PAMPA demonstrated high prediction capability.	[188]
**PVPA-based approaches**					
E80:_BSC_Cer:Chol:PA:ChS (77:23:0:0:0 or 50:27.5:12.5:7.5:2.5 wt%)	Mixed cellulose ester 650 nm filters	Permeation studies	Flu, IBP, IND, SAL, FITC-dextran, and CAL	PVPA approaches were able to distinguish between substances with different degrees of transdermal absorption.	[189]
E80:_BSC_Cer:Chol:PA:ChS (50:27.5:12.5:2.5:7.5 wt%)	Mixed cellulose ester 650 nm filters	Permeation and stability studies	Liposomal formulations of DCF	Adaptations to the original systems were performed towards nanoformulation testing. All liposomal formulations of DCF exhibited significantly higher permeabilities than DCF solution. PVPA has shown to be valuable for screening and the optimisation of lipid nanoformulations at the preformulation stage.	[190]
E80:_BSC_Cer:Chol:PA:ChS (77:23:0:0:0 or 50:27.5:12.5:7.5:2.5 wt%)	Mixed cellulose ester 650 nm filters	Stability and permeation studies	CAF, ACV, CPL, and CAL (liposomal formulation and/or aqueous solution)	PVPA systems were more promising in detecting permeability differences between aqueous solution and liposomal formulations than EpiSkin^®^. PVPA showed greater stability than EpiSkin^®^ (up to 2 weeks against 3-day testing window).	[191]
		Diffusion studies	CAF, DCF, CAP and CAL	SCS showed good correlation by ranking model drugs similarly to the ranks obtained using a pig ear skin model and were comparable to the literature on permeation through healthy human skin.	[192]
EPC/SPC:_BSC_Cer:Chol:PA:ChS (25–80:41–11:18–5: 3.75–1:11.25–3 wt%)	Nylon 450 nm filters	Menthol enhancer permeation studies, comparison with porcine ear skin and ATR-FTIR	FCA, PF, ALB, THC, and THP	PVPA model was adapted towards employment in a permeation-enhancing effect evaluation. The enhancement ratios obtained were in accordance with data from porcine ear skin. ATR-FTIR analysis detected a similar mechanism of menthol in both models (PVPA and porcine ear skin).	[193]
EPC:Chol (77:23 wt%)	Nylon 450 nm filters	SEM, electrical resistance, ATR-FTIR, and permeation studies	FCA, PF, ALB, THC, and THP	P_app_ values obtained through the PVPA-based system were well correlated with the values obtained through porcine ear skin. Better relevance between porcine ear skin and the PVPA-based system was found compared to that between porcine ear skin and a commercial Strat-M^®^ membrane.	[194]
Cer:EPC:Chol:SA:ChS (50:25:12.5:10:2.5 wt%)	Polycarbonate 400 nm filters	SEM, electrical resistance permeation, and stability studies	CAL, CAF, CSP, DCF, MTX, and NAP	Modified PVPA presented low permeability of hydrophilic markers when compared to the original PVPA (mimicking the intestinal barrier). Good data correlation with permeability studies in a pig ear model.	[195]
		Stability studies and permeability studies	CAL	Modified PVPA kept stable for 12 weeks when stored at −20 °C. Barrier integrity was not affected by the presence of a set of cosolvents, but ethanol altered its integrity.	[196]

_a_Cer—analog ceramides; AA—arachidic acid; ALB—albiflorin; APAP—acetaminophen; ATR-FTIR—attenuated total teflection Fourier-transform infrared; BA—behenic acid; BSC—bovine spinal cord; CA—cerotic acid; CAF—caffeine; CAL—calcein; CAP—chloramphenicol; Cer—ceramide; Cer[AdS]—α-hydroxy fatty acid/dihydrosphingosine base ceramide; Cer[AP]—α-hydroxy fatty acid/phytosphingosine base ceramide; Cer[EOdS]—ω-hydroxy fatty acid/dihydro-sphingosine base ceramide; Cer[EOS]—ester-linked ω-hydroxy fatty acid/sphingosine base ceramide; Cer[NdS]—non-hydroxy fatty acid/dihydrosphingosine base ceramide; Cer[NP]—non-hydroxy fatty acid/phytosphingosine base ceramide; Cer[NS]—non-hydroxy fatty acid/sphingosine base ceramide; Chol—cholesterol; ChS—cholesteryl sulfate; CIP—ciprofloxacin; CSP—cyclosporine; DCF—diclofenac; DDA—dodecyl acetate; DLS—dynamic light scattering; E-80—egg phospholipid lipoid E-80; EM—electron microscopy; EPC—egg phosphatidylcholine; ESEM—environmental scanning electron microscopy; FCA—ferulic acid; FFA—free fatty acid; Flu—flufenamic acid; FTIR—Fourier-transform infrared; FUR—furosemide; HC—hydrocortisone; _h_Cer—human isolated ceramide; IBP—ibuprofen; IND—indomethacin; IR—infrared; LA—lignoceric acid; MTX—methotrexate; NAP—naproxen; NFD—nifedipine; PA—palmitic acid; PABA—*p*-aminobenzoic acid; PAMPA—parallel artificial membrane permeability assay; PEF—pefloxacin; PF—paeoniflorin; PVPA—phospholipid vesicle-based permeation assay; _pig_Cer—pig isolated ceramide; SA—stearic acid; SAL—salicylic acid; SAXS—small-angle X-ray scattering; _syn_Cer—synthetic ceramide; SEM—scanning electron microscopy; SPC—soybean phosphatidylcholine; SWAXS—small- and wide-angle X-ray scattering; TA—tricosylic acid; TEWL—transepidermal water loss; TH—theophylline; THC—tetrahydrocolumbamine; THP—tetrahydropalmatine; WAXS—wide-angle X-ray scattering; VER—verapamil; XRPD—X-ray powder diffraction.

## Data Availability

Not applicable.

## Supplementary Materials

The following supporting information can be downloaded at https://www.mdpi.com/article/10.3390/pharmaceutics16060807/s1: Table S1. Examples of *Stratum Corneum* (SC) lipid model mixtures reported in the literature for either deciphering SC structure or as in vitro platforms to study compound-SC lipid matrix interaction (previous to 2010).
