# Supplementary material for: Lipid Biomimetic Models as Simple Yet Complex Tools to Predict Skin Permeation and Drug–Membrane Biophysical Interactions
Source: Pharmaceutics. 2024 Jun 14;16(6):807. doi: 10.3390/pharmaceutics16060807 (PMC11207520; doi:10.3390/pharmaceutics16060807)
Supplement: Supplementary file 1 [file pharmaceutics-16-00807-s001.zip › pharmaceutics-3028879-supplementary.pdf]

Supplementary Materials

Lipid Biomimetic Models as Simple Yet Complex Tools to Predict Skin Permeation and Drug–Membrane Biophysical Interactions

Eduarda Fernandes 1,*, Carla M. Lopes 2,3,4 and Marlene Lúcio 1,5,*

1 CF-UM-UP—Centro de Física das Universidades do Minho e Porto, Departamento de Física, Universidade do Minho, 4710-057 Braga, Portugal
2 FFP-I3ID—Instituto de Investigação, Inovação e Desenvolvimento, FP-BHS—Biomedical and Health Sciences Research Unit, Faculdade de Ciências da Saúde, Universidade Fernando Pessoa, 4200–150 Porto, Portugal; cmlopes@ufp.edu.pt
3 UCIBIO—Applied Molecular Biosciences Unit, MedTech–Laboratory of Pharmaceutical Technology, Faculty of Pharmacy, University of Porto, 4050-313 Porto, Portugal
4 Associate Laboratory i4HB, Institute for Health and Bioeconomy, Faculty of Pharmacy, University of Porto, 4050-313 Porto, Portugal
5 CBMA—Centro de Biologia Molecular e Ambiental, Departamento de Biologia, Universidade do Minho, 4710-057 Braga, Portugal
* Correspondence: eduardabfer@gmail.com (E.F.); mlucio@fisica.uminho.pt (M.L.)

Table S1. Examples of *Stratum Corneum* (SC) lipid model mixtures reported in the literature for either for deciphering SC structure or as in vitro platforms to study compound-SC lipid matrix interaction (previous to 2010).

Lipid Model Composition	Main Objective	Characterization techniques	Temperature (T) and pH conditions	Main Outcomes	Ref.
Cer[AP]:Chol:PA (1:0:1 or 1:0.01:1 molar ratio)	Monitor the SC lateral packing in absence and presence of permeation enhancers (Azone® and OA)	AFM	ND	· Chol reduced the driving force for minimizing phase separation, acting as a *lineactant*;<br>· Azone® induces reversed lipid phases, with less water content than the lamellar phase.	[1]
Cer[AP]:Chol:PA:ChS (55:25:15:5 wt%)	Evaluate the effect of permeation enhancers (urea, OA and 12G12) on SC system	SAXS	T: 30–90 °C<br>pH: ND	· SC system with two phases at 32 °C, which become mixed at ≈45 °C, and remained stable till 70 °C, when periodicity started to decrease;<br>· After cooling, one phase with two phase transitions at 45 and 70 °C is visible;<br>· While OA caused phase separation, urea and 12G12 showed a concentration-dependent shift in phase transition temperatures.	[2]

Lipid Model Composition	Main Objective	Characterization techniques	Temperature (T) and pH conditions	Main Outcomes	Ref.
	Prove the Chol position in the SC model	Neutron diffraction	T: 32 °C<br>pH: ND	· Chol is immersed in the hydrocarbon chain region of the bilayer.	[3]
	Investigate the structure and properties of SC vesicles	SANS	T: 32 and 60 °C<br>pH: 9.0	· At 32 °C, SC vesicles showed a bilayer thickness of ≈49 Å of which the hydrophobic region has a thickness of 27 Å;<br>· In excess of water, vesicular SC structure differs from partially hydrated planar SC bilayers;<br>· Chain-flip transitions are confirmed through the formation of cluster structures.	[4]
Cer[AP]:Chol:PA:ChS (Variable ratio)	Determine the internal membrane structure and water distribution across SC bilayer	Neutron diffraction	T: 32 and 82 °C<br>pH: ND	· Water layer with a thickness of ≈1 Å in full hydration SC model;<br>· SC model with 55:25:15:5 wt% at 32 °C, and 60 % humidity had lamellar repeated distances of 45.6 Å, while at 81 °C and 97 % of humidity was demixed into two phases with 45.8 Å and 40.5 Å;<br>· A decrease in Chol content tends to increase membrane thickness.	[5]
Cer[AP]:Chol:BA/CA:ChS (57:24:9.5:9.5 or 55:25:15:5 wt%)	Study the influence of FFA chain length on SC model assembly	Neutron diffraction	T: 20 °C<br>pH: ND	· Coexistence of FFA-rich phase and main SC phase, result of the decreased solubility of long-chained FFA within membrane created by Cer[AP];<br>· Long-chain FFA need to protrude into adjacent layer to fit in the phase sized by Cer[AP].	[6]
Cer[AP]:Chol:FFA:ChS FFA = SA, TA or CA (55:25:15:5 molar ratio) or FFA = BA (57:24:9.5:9.5 molar ratio)	Study the influence of FFA chain length on SC model assembly	Neutron diffraction	T: 20 °C<br>pH: ND	· An increase in FFA chain length decreased membrane repeated distance;<br>· Structure provided by Cer[AP] determines the stability of SC model;<br>· FFA incorporation results in the formation of two separated phases: main phase and FFA-rich phase.	[7]
Cer[AP]:PA:Chol:ChS (variable ratio)	Characterize the effects of Chol content on the SC membrane structure	DLS, SAXS, SANS, and molecular modelling	T: 31 and 85 °C<br>pH: 9	· Similar effect as in phospholipid systems: Chol decreases the order of well-ordered hydrocarbon chains below the SC main phase transition and increases chain order above the SC membrane main phase transition.	[8]
$_{BB}$Cer[NP]:Chol:PA (Variable ratio)	Characterize the polymorphism of SC lipids	IR spectroscopy	T: 10–80 °C<br>pH: 5.2	· Proportions of Cer and PA modulate the onset of the phase	[9]

Lipid Model Composition	Main Objective	Characterization techniques	Temperature (T) and pH conditions	Main Outcomes	Ref.
				transition and crystallinity of the model;<br>· Different ratios result in changes in phase behavior. However, in a general tendency, below 40 °C, lipids show very limited miscibility presenting crystalline domains; between 40–50 °C, a phase transition occurs to a liquid ordered phase;<br>· High Chol content leads to a liquid ordered phase stable up to 75 °C.	
hCer[NP]:Chol:FFA (1:1:1 molar ratio) FFA = OA:PA	Study the effect of lipid polymorphism on water permeation	SWAXS and water flux	T: 21 °C pH: ND	· Higher water flux through the solid crystalline SC phase compared to the liquid crystalline phases (lamellar and inverted hexagonal);<br>· Only minor difference in barrier capacity of SC were observed between lamellar and reversed hexagonal;<br>· Water flux through SC system decreased dramatically in the presence of Azone®, a permeation enhancer, which induces a transition from lamellar liquid crystalline phase into reversed micelles, possible through a cubic phase.	[10]
Cer[NP]:Chol:FFA (65:35:0 or 35:20:25 wt%) FFA = OA:PA:LOA (47:38:15 wt%)	Investigate the interaction of Span® 20 with SC lipid monolayers	Langmuir isotherms	T: 25 °C pH: ND	· Efficacy of a molecule as percutaneous enhancer can be determined by the correlation between its incorporation and the compressibility values of the corresponding monomolecular film;<br>· Span® 20 can be considered a permeation enhancer since its effects are not statistically different from Azone® effects.	[11]
Cer[NP]:Chol:PA (1:1:1 mol ratio)	Probe the molecular structure and explore the inter- and intramolecular interactions of SC components	Langmuir isotherms, IRRAS, and BAM	T: 21.5 °C pH: 5.5	· Chain packing and headgroup H-bonding in monolayers differ from those in bulk multilamellar SC system;<br>· Pure Cer monolayers revealed discrete and highly ordered crystalline domains, which upon FFA and Chol addition form a more fluid homogeneous monolayer.	[12]
Cer[NP]:Chol:PA Variable ratios	Evaluate the interest of ATR-FTIR and PCA	ATR-FTIR, PCA, and DSC	T: 25 °and 40–180 °C	· Relative importance of each component and the resulting lipid	[13]

Lipid Model Composition	Main Objective	Characterization techniques	Temperature (T) and pH conditions	Main Outcomes	Ref.
	coupling for studying lipid assembly within SC		pH: ND	assembly due to variable ratios were detected by PCA analysis of ATR-FTIR;<br>· The PCA method for analyzing ATR-FTIR spectra proved to be promising for understanding the effect of lipid composition on the structure of SC and for correlating lipid assembly and percutaneous permeation data.	
Cer[NP]/SPM:Chol:PA (1:1:1 molar ratio)	Investigate the mixing and crystallization properties of Cer with Chol and PA	FTIR	T: 10–70 °C<br>pH: 5.2	· While SPM forms a homogeneous liquid ordered mixture with Chol and PA, Cer[NP] mixture exhibits a rich polymorphism, transitioning from lipid mixing and the formation of orthorhombic solid crystalline domains at low temperatures to a homogeneous liquid crystalline phase at high temperatures;<br>· Cer[NP]-based model is more closely related to mouse and human SC;<br>· In SC mixture, while PA act as an enhancer of liquid crystalline structures, Chol promotes lipid mixing.	[14]
Cer[NS]:Chol/SA (1:1 molar ratio)	Understand the miscibility and intermolecular interactions of the SC model components	FTIR	T: 25–90 °C<br>pH: 5.5	· While Chol was miscible with Cer at physiological temperatures, SA was only miscible with Chol at relatively high temperatures (where FFA is disordered);<br>· In binary mixture of SA and Cer, a separate SA-rich phase persisted until at least 50 °C, whereas at higher temperatures the components appear to be quite miscible;<br>· Evidence of a complex interaction between SA and Cer, with high tendency for SA to associate with the Cer base chain;<br>· Evidence of the role of Chol in determining the miscibility characteristics of ternary SC mixtures.	[15]
Cer[NS]:Chol:PA (1:1:1 molar ratio)	Explore the phase behavior of SC lipids	^{2}H-NMR and vibrational spectroscopy	T: 25–80 °C<br>pH: 5.2	· Below 45 °C, the SC model showed coexistence of crystalline domains with a small fraction of lipids forming a liquid crystalline phase.	[16]

Lipid Model Composition	Main Objective	Characterization techniques	Temperature (T) and pH conditions	Main Outcomes	Ref.
	Determine the water permeability of a SC lipid model	QCM and GISAXS	T: 30 °C<br>pH: ND	Upon heating, a liquid ordered phase mainly composed of PA and Chol and a small fraction of Cer[NS] is formed;<br>· Cer's chain heterogeneity is a stronger criterion for lipid miscibility than hydrophobic chain matching.<br>· Presented approach showed to be a reliable measurement of water permeability of a SC membrane.	[17]
Cer[NS]:Chol:PA/SA (1:1:1 molar ratio)	Monitor the kinetics of raft formation through specific spectral parameters	FTIR	T: 25, 30, 35 °C<br>pH: 5.5	· The method based on changes in scissoring or rocking mode contours in IR spectra showed to be promising to study the domain formation kinetics;<br>· FFA-rich phase segregation kinetics were sensitive to quenching temperature and to chemical composition, and its determined timescales are like those observed in vivo when tracking restoration of permeability barrier.	[18]
Cer[AS]/Cer[NS]:Chol:PA (1:1:1 molar ratio)	Investigate the intermolecular interaction at headgroup and chain regions	FTIR	T: 20–80 °C<br>pH: 5.5	· Clear evidence of separated ordered domains of Cer and FFA, with chains packed in orthorhombic subcells at physiological temperatures for both SC models;<br>· Distinct H-bonding patterns were found depending on the type of Cer. Although intermolecular H-bonding between the headgroups are strong, they collapse upon the melting of Cer[NS]. Contrarily, in the case of Cer[AS], the strong headgroup H-bonding is still present between lateral domains even after conformational disordering of Cer.	[19]
Cer[AP]/Cer[NP]:Chol:SA (1:1:1 molar ratio)	Examine determinant vibrational modes revealing order conformation	FTIR	T: 16–116 °C<br>pH: 5.5	· Methylene stretching, rocking and bending revealed rotational freedom and hexagonal packing in both Cer and SA chains;<br>· When lipids were mixed in SC systems, they showed weaker H-bonding with FFA chains, presenting increased phase transition temperatures than when	[20]

Lipid Model Composition	Main Objective	Characterization techniques	Temperature (T) and pH conditions	Main Outcomes	Ref.
				each component was in its pure form.	
Cer[NP16]/Cer[NP24]: Chol:LA/PA (Variable ratio)	Investigate domain formation in SC monolayers	Langmuir isotherms, AFM, and SAXS	T: 19 °C<br>pH: 4.0	· Small amounts of Cer are miscible in the Chol-rich phase, with miscibility dependent on Cer chain length;<br>· Regularly shaped, spontaneous condensed Cer monolayer forms a solid crystalline phase, whereas a Chol-rich phase is considered to be less condensed;<br>· Several lipid phases coexist in SC model systems: lipids are spread into small domains of different compositions.	[21]
$_{h}$Cer[EOS]/$_{syn}$Cer[EOS]: Chol:FFA (1:1:1 molar ratio)<br>$_{syn}$Cer[EOS] = -*S*, -*O* or -*L*<br>FFA = PA:SA:BA:LA:CA (1:3:42:37:7 molar ratio)	Understand the role of Cer[EOS] in lipid phase behavior	SWAXS	T: RT<br>pH: 5.0	· Presence of Cer[EOS] is required for the formation of either LPP and a liquid crystalline phase: an optimal fraction of lipids forming a liquid crystalline phase seems to be a requisite for LPP formation;<br>· -S form-based system showed no LPP or liquid like phase; -O form resulted in a more prominent liquid crystalline phase than the -L form.	[22]
Cer[EOH]:SA (100-0:0-100 mol%)	Study the thermotropic phase behavior of the lipid mixture	FT-Raman spectroscopy and DSC	T: 40–90 °C<br>pH: ND	· Cer and SA showed to be immiscible with highly ordered structure in solid crystalline phase;<br>· In the mixture, Cer phase transition temperature decreases from 89°C to 63.5°C.	[23]
Cer[EOH]:OA (79-2:21-98 mol%)	Investigate the phase behavior of the lipid mixture	FT-Raman spectroscopy and DSC	T: -23–127 °C<br>pH: ND	· Cer and OA showed immiscibility in solid crystalline phase;<br>· Full hydration of the system caused a decrease in phase transition temperature; however, OA did not fluidize the Cer[EOH] structure.	[24]
$_{BB}$Cer[NP]/SPM:Chol: PA (1:1:1 molar ratio)	Characterize the SC model systems	^{2}H-NMR	20–75 °C<br>pH 5.2	· SPM-based system formed liquid-ordered membranes over a range of temperatures, with a maximum order parameter of ≈0.4 at 30 °C for positions C3-C10;<br>· Cer-based mixtures exhibited a complex polymorphism. Between 20–50 °C, a significant portion of membranes exist as a solid crystalline phase, with the remainder as liquid-ordered phase. Between 50–70 °C, membrane underwent a liquid-ordered	[25]

Lipid Model Composition	Main Objective	Characterization techniques	Temperature (T) and pH conditions	Main Outcomes	Ref.
				(anisotropic) to isotropic phase transition, which was reversible but with considerable hysteresis.	
$_{BB}$Cer[NP]/SPM:Chol:PA (1:1:1 or 2:0:1 molar ratio)	Examine the relationship between lipid composition and phase behavior	^{2}H-NMR	T: 20–75 °C pH: 5.2 to 7.4	· SPM-based system give rise to a stable liquid crystalline lamellar signal over temperature and pH range, whereas Cer-based model showed a temperature and pH dependent polymorphism;<br>· Cer-based system containing Chol showed phases' coexistence, liquid crystalline and solid crystalline, with this later being more ordered compared to typical solid crystalline phase. In the absence of Chol, the more ordered phase is pH dependent.	[26]
Cer/DPPC:Chol:PA :ChS (38 to 76:38 to 0:19:5 mol%)	Examine the interactions between SC main components	^{2}H-NMR and EM	T: 25–80 °C pH: ND	· DPPC-based systems showed no polymorphism throughout the temperature range studied, while Cer-based model revealed thermally-induced polymorphism.	[27]
Cer[EOS]/Cer[EOP]: Chol:FFA (1:1:1 molar ratio) FFA = PA:SA:AA:BA:TA:LA: CA (1.3:3.3:6.7:41.7:5.4:36.8 :4.7 molar ratio)	Investigate the effect of Cer headgroup architecture in the phase behavior of SC model	SAXS	T: RT pH: 5.0	· Cer[EOS] promotes the formation of LPP with 13 nm and its complete replacement by Cer[EOP] reduces formation of LPP and causes phase separation.	[28]
Cer[NP]/Cer[EOH]: DPPC:Chol:LLA: (1/0:2:1:1 molar ratio)	Investigate the effects of hyaluronan and its fragments on SC model after UV irradiation	DLS, TBA test, and EPR spectroscopy	ND	· Hyaluronan and hyaluronic acid fragments showed antioxidant effects by acting as iron chelators.	[29]
	Explore the redox mechanism of ascorbic acid	DLS, TBA test, and EPR spectroscopy	ND	· Ascorbic acid showed prooxidative effect dependent on concentration.	[30]
	Explore the antioxidant properties of metronidazole	TBA test	ND	· Metronidazole was able to protect LLA from UV-induced oxidation, acting as an antioxidant scavenger.	[31]
Cer:Chol:PA/LA (variable ratio) Cer = $_{pig}$Cer, $_{BB}$Cer, [NP16] or [NP24]	Investigate the phase behavior through Langmuir-Blodgett monolayers	Langmuir isotherms and AFM	ND	· Monolayer with each $_{pig}$Cer and $_{BB}$Cer in presence of Chol showed phase separation, however, increased Chol tends to improve miscibility, and mixtures with higher Chol content consisted of a single phase;<br>· FFA preferred mixing with Cer phase with similar chain length.	[32]

Lipid Model Composition	Main Objective	Characterization techniques	Temperature (T) and pH conditions	Main Outcomes	Ref.
$_{BB}$Cer:Chol:PA (1:1:1 or 2:2:1 molar ratio)	Interaction studies between OA and SC lipid model	NMR	T: 18–75 °C pH: 5.2	· Hypothesized a mechanism for permeation enhancer: OA extracts a fraction of SC membrane components, promoting phase separation in the system, which through reduced proportion of crystalline lipids, results in OA-rich domains with great permeability.	[33]
$_{pig}$Cer:Chol:FFA (1:1:0 or 2:1:0 or 1:1:1 molar ratio) FFA = CA:LA:BA:SA:PA (6.7:36.8:41.7:3.2:1.3 molar ratio)	Investigate the lateral packing of lipids in dry SC model	Cryo-electron diffraction	ND	· Presence of FFA caused a transition from a hexagonal to orthorhombic packing.	[34]
$_{BB}$Cer:Chol:LA (1:1:1 mol ratio) $_{h}$(Cer:Chol):FFA (1:0.9:0.4 mol ratio) FFA = AA:BA:LA:CA:MA: MeA (5:11:39:23:8:2 molar ratio)	Visualize the SC model structures (pH and temperature effect)	DSC, fluorescence spectroscopy, TPEF and laser confocal microscopies, DSC, and Laurdan GP	T: 10–95 °C pH: 5, 7 or 8	· Hydrated bilayers based on human SC extracts revealed a single solid crystalline phase at pH 7; the coexistence of different solid crystalline phases between pH 5 and 6, with micron-sized lipid domains; and no liquid crystalline phase at any pH;<br>· Lateral packing of BBCer-based system differs from that of human SC extract.	[35]
$_{BB}$Cer:Chol:PA (1:1:1 molar ratio)	Investigate the phase behavior of a SC model in freeze-dried and hydrated states	Raman spectroscopy	T: RT pH: 5.3	· While the freeze-dried SC model was homogeneous, a phase separation was clearly observed in the hydrated SC model with domains enriched in Chol, Cer or PA which were sized of few tens μm^2.	[36]
Cer:Chol:OA:LOA:PA: MyA:SA (44:25:10.7:4:11.9:1.2: 3.2 wt%) Cer = CeradermS®, [NP] or [AS]	Investigate the effect of different commercially available Cers on microstructure and physicochemical SC model	PLM, TEM, SWAXS, and DSC	T: 5–120 °C pH: 4.5 to 6	· Differences in thermal and optical behavior were detected for all tested models, depending on Cer component;<br>· Cer[AS]-based model was the most comparable with human SC lipids: a lamellar system with mosaic texture with oily streaks at RT and only one phase transition temperature at 59.7 °C.	[37]
$_{pig}$Cer:Chol:FFA (Variable ratio) FFA 1 = SA:PA:MyA:OA:LOA: POA (9.9:36.9:3.8:33.2:12.5: 3.6 mol%)	Examine the role of various lipid classes in a mimetic assembly of SC	SWAXS	T: 25–95 °C pH: 5	· Chol:Cer at 0.2 molar ratio is the minimum required to detect a LPP phase, while 0.4 to 1.0 molar ratios were considered the most similar to that found in intact pig SC (SPP=6.0 nm and LPP=13.2 nm);	[38]

Lipid Model Composition	Main Objective	Characterization techniques	Temperature (T) and pH conditions	Main Outcomes	Ref.
FFA 2 = PA:SA:BA:DA:LA:CA (1.3:3.2:41.7:5.4:36.8:6.9 mol%)				· Lipid phase behavior of Cer:Chol:FFA mixtures incorporating long-chain FFA closely mimics that of intact SC.	
Cer:Chol:PA:ChS (40:25:25:10 mol%)	Investigate the effects of transdermal absorption enhancers on barrier function of SC vesicular models	DSC	T: 20–120 °C pH: 7.5	· Vesicular models behaved as osmometers with excellent barrier function and phase transition temperatures similar to human SC lipids; · Pyrrolidone derivatives perturbed SC vesicles barrier function being incorporated into lipid layer and increasing system fluidity, which has a correlation with its transdermal absorption enhancing activity.	[39]
Cer[NP]:Chol:PA:ChS (Variable ratio)	Analyze the impact of mixtures based on Cer[NP] in creating a model that mimics the SC properties	TEM, TLC, PMR, and PCS	T: 37 °C pH: 7.5	· Formation of unilamellar vesicles in all lipid mixtures studied with reasonably homogeneous size distributions; · Increased concentrations of Cer, Chol and PA resulted in decreased size and internal volume of vesicles.	[40]
$_{h}$Cer:Chol:FFA (1:1:1 or 1:1:0.75 molar ratio) FFA = PA:SA:AA:BA:LA (1.3:3.3:7:47:41.4 wt%)	Study the thermotropic behavior of hydrated SC mixtures	IR spectroscopy	T: 20–100 °C pH: 5	· At RT, lower FFA content results in the coexistence of orthorhombic and hexagonal domains, while increased FFA levels to an equimolar mixture revealed dominant presence of orthorhombic lattice; at skin temperature, FFA-rich domains disappeared; · FFA contributes to the barrier properties of SC by enhancing formation of very densely packed orthorhombic lattice.	[41]
$_{pig}$Cer:Chol:PA (2:1:1, 2:2:1 or 1:0:1 molar ratio)	Determine the structural assembly of SC isolated lipids	SWAXS	T: ND pH: 7	· The characteristic lamellar repeating unit found in SC is not dependent on the presence of proteins, but it is dependent on the presence of specific Cers with appropriate concentrations of Chol and FFA; · LPP with 130 Å repeated distance is dependent on the presence of Cer[EOS].	[42]
$_{pig}$Cer:Chol:PA (2:1:1 molar ratio)	Investigate the assembly of SC model	SAXS	T: 20 °C pH: 6, 7 or 8.5	· Each repeating unit contained two asymmetric bilayers with a narrow fluid space on one side of the	[43]

Lipid Model Composition	Main Objective	Characterization techniques	Temperature (T) and pH conditions	Main Outcomes	Ref.
				bilayer that increased with increasing pH due to electrostatic repulsion between bilayers because of PA ionization.	
$_{BB}$Cer:Chol:FFA:ChS (55:25:15:5 wt%) FFA = LA:OA:PA (1:2:1 wt ratio)	Characterize the SC vesicles	CryoEM, QELS, EPR, and DSC	T: 5–90 °C pH: 6, 7.4 or 9	· SC vesicles were characterized as spherical, revealing a narrow size distribution of ≈119 nm and a broad endothermic phase transition near 60–65 °C;<br>· Vesicles were stable for several weeks at pH 9 and 15 –24 h at pH 6;<br>· EPR studies revealed that motion is more restricted near the polar headgroup region than near the center of alkyl chain region.	[44]
$_{h}$Cer:Chol:FFA:ChS (Variable ratio) FFA = PA:SA:BA:LA:CA (1:3:42:37:7 molar ratio)	Examine the phase behavior of $_{h}$Cer-based SC mixtures	SWAXS	T: 25–90 °C pH: 5 or 7.4	· Addition of FFA to the SC system promoted the formation of SPP and favored the orthorhombic lattice, while also promotes the formation of a liquid like phase;<br>· LPP phase dominates in hCer:Chol system, whereas SPP is dominant when Cer[EOS] is lacking.	[45]
$_{pig}$Cer:Chol:FFA (Variable ratio) FFA = PA:SA:BA:LA:CA (1.3:3.2:41.7:36.8:6.7 molar ratio)	Investigate the role of Cer[EOS] in SC lipid lamellar structure	SWAXS	T: ND pH: 5	· A model based on Cer[EOS] plays a dominant role in LPP formation and is purposed for the molecular assembly of the two lamellar phases: SPP composed of one bilayer and LPP containing two regions, one formed by partly interdigitating Cers with long-chain FFA (24–26C), and another made of interdigitating Cers with shorter chained FFAs (16–18C).	[46]
Cer:Chol:FFA (1:1:1 molar ratio) Cer = $_{BB}$[NP], $_{BB}$[EOH], [NP16], [NP24], [EOS]:$_{BB}$[NP], [EOS]:$_{BB}$[EOH] or [EOS]:[NP24] FFA = PA:SA:AA:BA:DA:LA:CA (1.3:3.3:6.7:41.7:5.4:36.8:4.7 molar ratio)	Examine the lamellar and lateral packing of $_{syn}$Cers in SC model	SWAXS	T: ND pH: 5	· $_{BB}$[NP]- and $_{BB}$[EOH]-based mixtures did not form LPP, and the authors considered that $_{BB}$[NP]-based mixture cannot represent the SC lipid structure;<br>· The mixture containing [EOS]:[NP24] mimicked intact SC to a certain extent, since LPP presented a repeated distance of 11.6 nm.	[47]
Cer[NP]:Cer[EOH]: DPPC:Chol (0:0:2:1 or 1:1:2:1 molar ratio)	Investigate the effects of ascorbic acid, iron ions and UV irradiation on SC model	EPR spectroscopy and TBA test	T: 30 °C pH: ND	· A new mechanism of lipid damage by ascorbic acid and transition metal ions after UV exposure was determined, transcending simple	[48]

Lipid Model Composition	Main Objective	Characterization techniques	Temperature (T) and pH conditions	Main Outcomes	Ref.
				redox behavior and transition metal interaction.	
Cer[EOS]:Cer[AP]: Chol:PA:ChS (Variable ratio)	Investigate the effect of Cer[EOS] in LPP formation	Neutron diffraction	T: 32 °C pH: 5.5	· Under experimental conditions, no LPP was detected in any of the tested molar ratios; · A Cer[EOS] assembly is proposed: these molecules permeate from one membrane layer into an adjacent layer inside a phase with a repeated distance of 45.2 Å, predominantly formed by Cer[AP].	[49]
Cer[EOS]:Cer[AP]: Chol:FFA (23:10:33:33 wt%) FFA = PA, BA, TA or CA	Investigate the nanostructure of a SC model	Neutron diffraction	T: 32 °C pH: ND	· Cer[EOS] can assemble into a Cer[AP]-based SPP by spanning through whole bilayer and extending into adjacent bilayer.	[50]
Cer[EOS]:Cer[EOH]: Cer[NP]:Chol:FFA (Variable ratio) FFA = PA:SA:AA:BA:TA:LA: CA (1.3:3.3:6.7:41.7:5.4:36.8 :4.7 molar ratio)	Determine the optical molar ratio of Cers for the formation of LPP	SAXS	T: 80 °C pH: 5.0	· LPP was detected in all SC mixture variations, however, [EOS]:[EOH] ratio affects the formation of LPP, and the optimal ratio depends on the presence of FFA; · In the absence of FFA, a [EOS]:[EOH]:[NP] molar ratio of 1:5:4 is optimal for LPP formation, while in the presence of FFA, the optimal Cer content shifts to 1:7:2.	[51]
Cer[EOS]:Cer[NP]:Cer [EOH]:Chol (0.1:0.7:0.2:1 molar ratio) FFA = PA:SA:AA:BA:MyA: LA:CA (1.3:3.3:6.7:41.7:5.4:36.8 :4.7 molar ratio)	Examine the role of each lipid class in the formation of a SC barrier	SWAXS	T: 25–95 °C pH: 5.0	· SC mixtures based on synthetic Cers presented two characteristic lamellar phases similar to native SC; · Composition and temperature of lipid mixtures play crucial roles in LPP formation; · FFA have great influence on phase behavior of mixture: improves Chol and Cers miscibility, increases ordering of both LPP and SPP structures and is the main promoter of orthorhombic packing.	[52]
Cer:Chol (1:1 molar ratio) Cer = [EOS]:[NS]:[NP]: [EOH]:[AS]:[AP] (Variable ratio)	Study Cers role in the SC lipid assembly	SAXS	ND pH: 5.0	· At Cer:Chol equimolar ratio, the lamellar assembly is the least sensitive to Cer composition, whereas at increased Cer:Chol ratio, Cer composition plays a prominent role in lamellar phase assembly.	[53]
Cer:Chol:FFA (1:1:0 or 1:1:1 molar ratio)	Examine the lipid assembly of SC mixtures	FTIR and SAXS	T: 20–100°C pH: 5.0	· Cer headgroup assembly affects lateral packing and conformational ordering of SC mixtures;	[54]

Lipid Model Composition	Main Objective	Characterization techniques	Temperature (T) and pH conditions	Main Outcomes	Ref.
Cer = [EOS]:[NS]:[NP24]:[EOH]:[NP16]:[AP] (15:51:16:4:9:5 mol%) FFA= PA:SA:AA:BA:LA (1.8:4:7.6:47.8:38.9 mol%)				· Major lipid fraction forms a solid crystalline packing, while linoleate moiety of acylCers participates in a liquid crystalline phase.	
Cer:Chol:FFA (1:1:1 or 2:1:1) Cer = $_{pig}$Cer, [EOS]:$_{h}$Cer or [EOS]:[NS]:[NP]:[EOH]:[NP16]:[AP] (variable ratio) FFA = PA:SA:AA:BA:TA:LA:CA (1.8:4:7.7:42.6:5.2:34.7:4.1 molar ratio)	Examine the molecular assembly of LPP of a SC model	SAXS	T: ND pH: 5.0	· SC lipid mixture revealed a lamellar phase with repeated distances from 12.1 to 13.8 nm;<br>· Electron density distribution in the LPP of the mixtures resembled the LPP from isolated SC.	[55]
Cer:Chol:FFA Cer = [EOS], [NS], [NP24], [AS], [NP16], [AP] and [EOP] (variable combinations at variable ratios) FFA = PA:SA:AA:BA:TA:LA:CA (1.3:3.3:6.7:41.7:5.4:36.8:4.7 molar ratio)	Examine the assembly in SC models with well-defined Cer mixtures varying in headgroup architecture and acyl chain length	SWAXS	T: 25–95°C pH: 5.0	· For a proper lipid assembly, a certain chain length variation should be present in the mixture, either in the FFA fraction or in the Cer fraction;<br>· Cer[EOS] promoted LPP formation more efficiently than Cer[EOP];<br>· SC lipid mixtures with well-defined synthetic Cers can closely mimic lamellar and lateral packing of native SC.	[56]
Cer[NP]:Chol:SA (Variable ratio)	Investigate the interaction between components in SC model	DSC and SWAXS	T: 30–140 °C pH: ND	· In the lipid bilayer of binary mixture, a homogeneous distribution of Cer[NP] and SA with hydrocarbon chains lying perpendicular to the surface was described;<br>· Ternary mixture resulted in a pseudo-hexagonal structure, even in the solid crystalline phase, with all components showing miscibility and the hydrocarbon chains lying perpendicular to the bilayer surface.	[57]

AA – arachidonic acid; AFM – atomic force microscopy; ATR-FTIR – attenuated total reflection Fourier-transform infrared spectroscopy; BA – behenic acid; BAM – Brewster angle microscopy; $_{BB}$Cer – bovine brain ceramide; $_{br}$Cer – branched ceramide; CA – cerotic acid; Cer – ceramide; Cer[AdS] – α-hydroxy fatty acid/dihydrosphingosine base ceramide; Cer[AP] – α-hydroxy fatty acid/phytosphingosine base ceramide; Cer[EOS] – ester-linked ω-hydroxy fatty acid/sphingosine base ceramide; Cer[NdS] – non-hydroxy fatty acid/dihydrosphingosine base ceramide; Cer[NH] – non-hydroxy fatty acid/6-hydroxy-sphingosine base ceramide; Cer[NP] – non-hydroxy fatty acid/phytosphingosine base ceramide; Cer[NS] – non-hydroxy fatty acid/sphingosine base ceramide; Chol – cholesterol; ChS – cholesteryl sulfate; CPE – chemical permeation enhancer; DA –

docosatrienoic acid; DLS – dynamic light scattering; DMSO – dimethyl sulfoxide; DPPC – dipalmitoylphosphatidylcholine; DSC – differential scanning calorimetry; EIS – electrochemical impedance spectroscopy; EM – electron microscopy; EPR – electron paramagnetic resonance; FFA – free fatty acid; FRET – Förster resonance energy transfer; FT – Fourier-transform; FTIR – Fourier-transform infrared spectroscopy; GISAXS – Grazing incidence small-angle X-ray scattering; GIXD – Grazing incidence X-ray diffraction; $_{h}$Cer – human isolated Ceramide; IPM – isopropyl myristate; IR – infrared; IRRAS – infrared reflection absorption spectroscopy; ITC – isothermal titration calorimetry; LA – lignoceric acid; LLA – α-linolenic acid; LOA – linoleic acid; LPP – long periodicity phase; MA – montanic acid; MBA - N′-methylenebisacrylamide; MD – molecular dynamics; MeA – melissic acid; MyA – myristic acid; ND – not discriminated; NIPAM - n-isopropylacrylamide; NMR – nuclear magnetic resonance; OA – oleic acid; PA – palmitic acid; PCA – principal component analysis; PCS – photon correlation spectroscopy; $_{pig}$Cer – pig isolated ceramide; PLM – polarized light microscopy; PMR – proton magnetic resonance; POA – palmitoleic acid; QCM – quartz-crystal microbalance; QELS – quasi-elastic light scattering; RT- room temperature; SA – stearic acid; SANS – small-angle neutron scattering; SAXS – small-angle X-ray scattering, SWAXS – small and wide angle X-ray scattering; SC – *Stratum corneum*; SP – Sphingosine; SPP – short periodicity phase; TA – tricosylic acid; TBA – thiobarbituric acid; TEM – transmission electron microscopy; TLC – thin-layer chromatography; TPEF – two-photon excitation fluorescence; UV – ultraviolet; WAXS – wide-angle X-ray scattering; wt – weight.

References

1. Engstrom, S.; Ekelund, K.; Engblom, J.; Eriksson, L.; Sparr, E.; Wennerstrom, H. The skin barrier from a lipid perspective. *Acta Derm.-Venereol., Suppl.* **2000**, *208*, 31-35, doi:10.1080/000155500750042835.
2. Zbytovská, J.; Vávrová, K.; Kiselev, M.A.; Lessieur, P.; Wartewig, S.; Neubert, R.H.H. The effects of transdermal permeation enhancers on thermotropic phase behaviour of a stratum corneum lipid model. *Colloids Surf., A* **2009**, *351*, 30-37, doi:10.1016/j.colsurfa.2009.09.025.
3. Kessner, D.; Kiselev, M.A.; Hauss, T.; Dante, S.; Wartewig, S.; Neubert, R.H. Localisation of partially deuterated cholesterol in quaternary SC lipid model membranes: a neutron diffraction study. *Eur. Biophys. J.* **2008**, *37*, 1051-1057, doi:10.1007/s00249-008-0265-4.
4. Zemlyanaya, E.V.; Kiselev, M.A.; Neubert, R.; Kohlbrecher, J.; Aksenov, V.L. Investigation of the structure and properties of model membranes of the stratum corneum by small-angle neutron scattering. *J. Surf. Invest.: X-Ray, Synchrotron Neutron Tech.* **2008**, *2*, 884-889, doi:10.1134/s1027451008060104.
5. Kiselev, M.A.; Ryabova, N.Y.; Balagurov, A.M.; Dante, S.; Hauss, T.; Zbytovska, J.; Wartewig, S.; Neubert, R.H. New insights into the structure and hydration of a stratum corneum lipid model membrane by neutron diffraction. *Eur. Biophys. J.* **2005**, *34*, 1030-1040, doi:10.1007/s00249-005-0488-6.
6. Schroeter, A.; Kiselev, M.A.; Hauss, T.; Dante, S.; Neubert, R.H. Evidence of free fatty acid interdigitation in stratum corneum model membranes based on ceramide [AP] by deuterium labelling. *Biochim. Biophys. Acta* **2009**, *1788*, 2194-2203, doi:10.1016/j.bbamem.2009.07.024.
7. Ruettinger, A.; Kiselev, M.A.; Hauss, T.; Dante, S.; Balagurov, A.M.; Neubert, R.H. Fatty acid interdigitation in stratum corneum model membranes: a neutron diffraction study. *Eur. Biophys. J.* **2008**, *37*, 759-771, doi:10.1007/s00249-008-0258-3.
8. Zbytovská, J.; Kiselev, M.A.; Funari, S.S.; Garamus, V.M.; Wartewig, S.; Palát, K.; Neubert, R. Influence of cholesterol on the structure of stratum corneum lipid model membrane. *Colloids Surf., A* **2008**, *328*, 90-99, doi:10.1016/j.colsurfa.2008.06.032.
9. Velkova, V.; Lafleur, M. Influence of the lipid composition on the organization of skin lipid model mixtures: an infrared spectroscopy investigation. *Chem. Phys. Lipids* **2002**, *117*, 63-74, doi:10.1016/s0009-3084(02)00042-7.
10. Norlen, L.; Engblom, J. Structure-related aspects on water diffusivity in fatty acid-soap and skin lipid model systems. *J. Controlled Release* **2000**, *63*, 213-226, doi:10.1016/s0168-3659(99)00201-1.

11. Lopez-Castellano, A.; Cortell-Ivars, C.; Lopez-Carballo, G.; Herraez-Dominguez, M. The influence of Span20 on stratum corneum lipids in langmuir monolayers: comparison with Azone. *Int. J. Pharm.* **2000**, *203*, 245-253, doi:10.1016/s0378-5173(00)00463-4.
12. Flach, C.R.; Mendelsohn, R.; Rerek, M.E.; Moore, D.J. Biophysical studies of model stratum corneum lipid monolayers by infrared reflection-absorption spectroscopy and Brewster angle microscopy. *J. Phys. Chem. B* **2000**, *104*, 2159-2165, doi:DOI 10.1021/jp9936805.
13. Laugel, C.; Yagoubi, N.; Baillet, A. ATR-FTIR spectroscopy: a chemometric approach for studying the lipid organisation of the stratum corneum. *Chem. Phys. Lipids* **2005**, *135*, 55-68, doi:10.1016/j.chemphyslip.2005.02.001.
14. Lafleur, M. Phase behaviour of model stratum corneum lipid mixtures: an infrared spectroscopy investigation. *Can. J. Chem.* **1998**, *76*, 1501-1511, doi:10.1139/v98-114.
15. Chen, H.; Mendelsohn, R.; Rerek, M.E.; Moore, D.J. Effect of cholesterol on miscibility and phase behavior in binary mixtures with synthetic ceramide 2 and octadecanoic acid. Infrared studies. *Biochim. Biophys. Acta* **2001**, *1512*, 345-356, doi:10.1016/s0005-2736(01)00339-x.
16. Brief, E.; Kwak, S.; Cheng, J.T.; Kitson, N.; Thewalt, J.; Lafleur, M. Phase behavior of an equimolar mixture of N-palmitoyl-D-erythro-sphingosine, cholesterol, and palmitic acid, a mixture with optimized hydrophobic matching. *Langmuir* **2009**, *25*, 7523-7532, doi:10.1021/la9003643.
17. Lee, D.; Ashcraft, J.N.; Verploegen, E.; Pashkovski, E.; Weitz, D.A. Permeability of model stratum corneum lipid membrane measured using quartz crystal microbalance. *Langmuir* **2009**, *25*, 5762-5766, doi:10.1021/la804105t.
18. Moore, D.J.; Snyder, R.G.; Rerek, M.E.; Mendelsohn, R. Kinetics of membrane raft formation: fatty acid domains in stratum corneum lipid models. *J. Phys. Chem. B* **2006**, *110*, 2378-2386, doi:10.1021/jp054875h.
19. Moore, D.J.; Rerek, M.E. Insights into the molecular organization of lipids in the skin barrier from infrared spectroscopy studies of stratum corneum lipid models. *Acta Derm.-Venereol., Suppl.* **2000**, *208*, 16-22, doi:10.1080/000155500750042817.
20. Rerek, M.E.; Van Wyck, D.; Mendelsohn, R.; Moore, D.J. FTIR spectroscopic studies of lipid dynamics in phytosphingosine ceramide models of the stratum corneum lipid matrix. *Chem. Phys. Lipids* **2005**, *134*, 51-58, doi:10.1016/j.chemphyslip.2004.12.002.
21. Sparr, E.; Eriksson, L.; Bouwstra, J.A.; Ekelund, K. AFM Study of Lipid Monolayers: III. Phase Behavior of Ceramides, Cholesterol and Fatty Acids. *Langmuir* **2000**, *17*, 164-172, doi:10.1021/la000271n.
22. Bouwstra, J.A.; Gooris, G.S.; Dubbelaar, F.E.; Ponec, M. Phase behavior of stratum corneum lipid mixtures based on human ceramides: the role of natural and synthetic ceramide 1. *J. Invest. Dermatol.* **2002**, *118*, 606-617, doi:10.1046/j.1523-1747.2002.01706.x.
23. Neubert, R.; Rettig, W.; Wartewig, S.; Wegener, M.; Wienhold, A. Structure of stratum corneum lipids characterized by FT-Raman spectroscopy and DSC. II. Mixtures of ceramides and saturated fatty acids. *Chem. Phys. Lipids* **1997**, *89*, 3-14, doi:10.1016/s0009-3084(97)00049-2.
24. Wartewig, S.; Neubert, R.; Rettig, W.; Hesse, K. Structure of stratum corneum lipids characterized by FT-Raman spectroscopy and DSC. IV. Mixtures of ceramides and oleic acid. *Chem. Phys. Lipids* **1998**, *91*, 145-152, doi:10.1016/s0009-3084(97)00105-9.
25. Fenske, D.B.; Thewalt, J.L.; Bloom, M.; Kitson, N. Models of stratum corneum intercellular membranes: 2H NMR of macroscopically oriented multilayers. *Biophys. J.* **1994**, *67*, 1562-1573, doi:10.1016/S0006-3495(94)80629-0.
26. Kitson, N.; Thewalt, J.; Lafleur, M.; Bloom, M. A model membrane approach to the epidermal permeability barrier. *Biochemistry* **1994**, *33*, 6707-6715, doi:10.1021/bi00187a042.

27. Abraham, W.; Downing, D.T. Deuterium NMR investigation of polymorphism in stratum corneum lipids. *Biochim. Biophys. Acta* **1991**, *1068*, 189-194, doi:10.1016/0005-2736(91)90209-q.
28. de Jager, M.; Gooris, G.; Ponec, M.; Bouwstra, J. Acylceramide head group architecture affects lipid organization in synthetic ceramide mixtures. *J. Invest. Dermatol.* **2004**, *123*, 911-916, doi:10.1111/j.0022-202X.2004.23477.x.
29. Trommer, H.; Wartewig, S.; Bottcher, R.; Poppl, A.; Hoentsch, J.; Ozegowski, J.H.; Neubert, R.H. The effects of hyaluronan and its fragments on lipid models exposed to UV irradiation. *Int. J. Pharm.* **2003**, *254*, 223-234, doi:10.1016/s0378-5173(03)00033-4.
30. Trommer, H.; Bottcher, R.; Poppl, A.; Hoentsch, J.; Wartewig, S.; Neubert, R.H. Role of ascorbic acid in stratum corneum lipid models exposed to UV irradiation. *Pharm. Res.* **2002**, *19*, 982-990, doi:10.1023/a:1016414422106.
31. Narayanan, S.; Hunerbein, A.; Getie, M.; Jackel, A.; Neubert, R.H. Scavenging properties of metronidazole on free oxygen radicals in a skin lipid model system. *J. Pharm. Pharmacol.* **2007**, *59*, 1125-1130, doi:10.1211/jpp.59.8.0010.
32. ten Grotenhuis, E.; Demel, R.A.; Ponec, M.; Boer, D.R.; van Miltenburg, J.C.; Bouwstra, J.A. Phase behavior of stratum corneum lipids in mixed Langmuir-Blodgett monolayers. *Biophys. J.* **1996**, *71*, 1389-1399, doi:10.1016/S0006-3495(96)79341-4.
33. Rowat, A.C.; Kitson, N.; Thewalt, J.L. Interactions of oleic acid and model stratum corneum membranes as seen by 2H NMR. *Int. J. Pharm.* **2006**, *307*, 225-231, doi:10.1016/j.ijpharm.2005.10.008.
34. Pilgram, G.S.K.; Pelt, A.M.E.-v.; Oostergetel, G.T.; Koerten, H.K.; Bouwstra, J.A. Study on the lipid organization of stratum corneum lipid models by (cryo-) electron diffraction. *J. Lipid Res.* **1998**, *39*, 1669-1676, doi:10.1016/s0022-2275(20)32197-0.
35. Plasencia, I.; Norlen, L.; Bagatolli, L.A. Direct visualization of lipid domains in human skin stratum corneum's lipid membranes: effect of pH and temperature. *Biophys. J.* **2007**, *93*, 3142-3155, doi:10.1529/biophysj.106.096164.
36. Percot, A.; Lafleur, M. Direct observation of domains in model stratum corneum lipid mixtures by Raman microspectroscopy. *Biophys. J.* **2001**, *81*, 2144-2153, doi:10.1016/S0006-3495(01)75862-6.
37. Glombitza, B.; Muller-Goymann, C.C. Influence of different ceramides on the structure of in vitro model lipid systems of the stratum corneum lipid matrix. *Chem. Phys. Lipids* **2002**, *117*, 29-44, doi:10.1016/s0009-3084(02)00029-4.
38. Bouwstra, J.A.; Gooris, G.S.; Cheng, K.; Weerheim, A.; Bras, W.; Ponec, M. Phase behavior of isolated skin lipids. *J. Lipid Res.* **1996**, *37*, 999-1011, doi:10.1016/s0022-2275(20)42010-3.
39. Kim, C.-K.; Hong, M.-S.; Kim, Y.-B.; Han, S.-K. Effect of penetration enhancers (pyrrolidone derivatives) on multilamellar liposomes of stratum corneum lipid: a study by UV spectroscopy and differential scanning calorimetry. *Int. J. Pharm.* **1993**, *95*, 43-50, doi:10.1016/0378-5173(93)90388-v.
40. de la Maza, A.; Manich, A.M.; Coderch, L.; Bosch, P.; Parra, J.L. The formation of liposomes in vitro by mixtures of lipids modeling the composition of the stratum corneum. *Colloids Surf., A* **1995**, *101*, 9-19, doi:10.1016/0927-7757(95)03158-a.
41. Gooris, G.S.; Bouwstra, J.A. Infrared spectroscopic study of stratum corneum model membranes prepared from human ceramides, cholesterol, and fatty acids. *Biophys. J.* **2007**, *92*, 2785-2795, doi:10.1529/biophysj.106.094292.
42. McIntosh, T.J.; Stewart, M.E.; Downing, D.T. X-ray diffraction analysis of isolated skin lipids: reconstitution of intercellular lipid domains. *Biochemistry* **1996**, *35*, 3649-3653, doi:10.1021/bi952762q.
43. McIntosh, T.J. Organization of skin stratum corneum extracellular lamellae: diffraction evidence for asymmetric distribution of cholesterol. *Biophys. J.* **2003**, *85*, 1675-1681, doi:10.1016/S0006-3495(03)74597-4.

44. Hatfield, R.M.; Fung, L.W. Molecular properties of a stratum corneum model lipid system: large unilamellar vesicles. *Biophys. J.* **1995**, *68*, 196-207, doi:10.1016/S0006-3495(95)80175-X.
45. Bouwstra, J.A.; Gooris, G.S.; Dubbelaar, F.E.R.; Ponec, M. Phase behavior of lipid mixtures based on human ceramides: coexistence of crystalline and liquid phases. *J. Lipid Res.* **2001**, *42*, 1759-1770, doi:10.1016/s0022-2275(20)31502-9.
46. Bouwstra, J.A.; Gooris, G.S.; Dubbelaar, F.E.R.; Weerheim, A.M.; Ijzerman, A.P.; Ponec, M. Role of ceramide 1 in the molecular organization of the stratum corneum lipids. *J. Lipid Res.* **1998**, *39*, 186-196, doi:10.1016/s0022-2275(20)34214-0.
47. de Jager, M.W.; Gooris, G.S.; Dolbnya, I.P.; Bras, W.; Ponec, M.; Bouwstra, J.A. The phase behaviour of skin lipid mixtures based on synthetic ceramides. *Chem. Phys. Lipids* **2003**, *124*, 123-134, doi:10.1016/s0009-3084(03)00050-1.
48. Trommer, H.; Bottcher, R.; Huschka, C.; Wohlrab, W.; Neubert, R.H. Further investigations on the role of ascorbic acid in stratum corneum lipid models after UV exposure. *J. Pharm. Pharmacol.* **2005**, *57*, 963-972, doi:10.1211/0022357056703.
49. Kessner, D.; Kiselev, M.; Dante, S.; Hauss, T.; Lersch, P.; Wartewig, S.; Neubert, R.H. Arrangement of ceramide [EOS] in a stratum corneum lipid model matrix: new aspects revealed by neutron diffraction studies. *Eur. Biophys. J.* **2008**, *37*, 989-999, doi:10.1007/s00249-008-0328-6.
50. Schroter, A.; Kessner, D.; Kiselev, M.A.; Hauss, T.; Dante, S.; Neubert, R.H. Basic nanostructure of stratum corneum lipid matrices based on ceramides [EOS] and [AP]: a neutron diffraction study. *Biophys. J.* **2009**, *97*, 1104-1114, doi:10.1016/j.bpj.2009.05.041.
51. de Jager, M.W.; Gooris, G.S.; Dolbnya, I.P.; Ponec, M.; Bouwstra, J.A. Modelling the stratum corneum lipid organisation with synthetic lipid mixtures: the importance of synthetic ceramide composition. *Biochim. Biophys. Acta* **2004**, *1664*, 132-140, doi:10.1016/j.bbamem.2004.05.001.
52. de Jager, M.W.; Gooris, G.S.; Dolbnya, I.P.; Bras, W.; Ponec, M.; Bouwstra, J.A. Novel lipid mixtures based on synthetic ceramides reproduce the unique stratum corneum lipid organization. *J. Lipid Res.* **2004**, *45*, 923-932, doi:10.1194/jlr.M300484-JLR200.
53. Bouwstra, J.A.; Dubbelaar, F.E.; Gooris, G.S.; Weerheim, A.M.; Ponec, M. The role of ceramide composition in the lipid organisation of the skin barrier. *Biochim. Biophys. Acta* **1999**, *1419*, 127-136, doi:10.1016/s0005-2736(99)00057-7.
54. Janssens, M.; Gooris, G.S.; Bouwstra, J.A. Infrared spectroscopy studies of mixtures prepared with synthetic ceramides varying in head group architecture: coexistence of liquid and crystalline phases. *Biochim. Biophys. Acta* **2009**, *1788*, 732-742, doi:10.1016/j.bbamem.2009.01.003.
55. Groen, D.; Gooris, G.S.; Bouwstra, J.A. New insights into the stratum corneum lipid organization by X-ray diffraction analysis. *Biophys. J.* **2009**, *97*, 2242-2249, doi:10.1016/j.bpj.2009.07.040.
56. de Jager, M.W.; Gooris, G.S.; Ponec, M.; Bouwstra, J.A. Lipid mixtures prepared with well-defined synthetic ceramides closely mimic the unique stratum corneum lipid phase behavior. *J. Lipid Res.* **2005**, *46*, 2649-2656, doi:10.1194/jlr.M500221-JLR200.
57. Ohta, N.; Hatta, I. Interaction among molecules in mixtures of ceramide/stearic acid, ceramide/cholesterol and ceramide/stearic acid/cholesterol. *Chem. Phys. Lipids* **2002**, *115*, 93-105, doi:10.1016/s0009-3084(02)00027-0.
58. de Jager, M.; Groenink, W.; van der Spek, J.; Janmaat, C.; Gooris, G.; Ponec, M.; Bouwstra, J. Preparation and characterization of a stratum corneum substitute for in vitro percutaneous penetration studies. *Biochim. Biophys. Acta* **2006**, *1758*, 636-644, doi:10.1016/j.bbamem.2006.04.001.
59. de Jager, M.; Groenink, W.; Bielsa i Guivernau, R.; Andersson, E.; Angelova, N.; Ponec, M.; Bouwstra, J. A novel in vitro percutaneous penetration model: evaluation of barrier

properties with p-aminobenzoic acid and two of its derivatives. *Pharm. Res.* **2006**, *23*, 951-960, doi:10.1007/s11095-006-9909-1.
60. Groen, D.; Gooris, G.S.; Ponec, M.; Bouwstra, J.A. Two new methods for preparing a unique stratum corneum substitute. *Biochim. Biophys. Acta* **2008**, *1778*, 2421-2429, doi:10.1016/j.bbamem.2008.06.015.
61. Sinko, B.; Kokosi, J.; Avdeef, A.; Takacs-Novak, K. A PAMPA study of the permeability-enhancing effect of new ceramide analogues. *Chem. Biodiversity* **2009**, *6*, 1867-1874, doi:10.1002/cbdv.200900149.
